# Filtering the noise: a cerebellar-centered framework for understanding and treating mental illness -a paradigm shift in psychiatry

**DOI:** 10.3389/fpsyt.2026.1772265

**Published:** 2026-04-13

**Authors:** Craig F. Ferris

**Affiliations:** Departments of Psychology and Pharmaceutical Science, Center for Translational Neuroimaging, Northeastern University, Boston, MA, United States

**Keywords:** evolution, P50, prediction theory, prepulse inhibition, schizophrenia

## Abstract

For the past half-century, psychiatric drug development has largely focused on tweaking neurotransmitter receptors and chemical pathways. Yet despite billions of dollars invested and major advances in neuroscience, truly innovative treatments for mental illness remain scarce. Disorders like depression, schizophrenia, and post-traumatic stress disorder (PTSD) continue to be managed with drugs discovered decades ago that often provide only partial relief, with remission rates of approximately 30-40% for treatment-resistant depression and 60-70% of schizophrenia patients experiencing persistent symptoms despite medication. This stagnation has prompted a paradigm shift - what if the key to treating mental illness is not just which receptor a drug targets, but how it changes the brain’s processing of sensory information? In this treatise, I propose that many psychiatric conditions stem from breakdowns in the brain’s sensory filtering mechanisms, the neural circuits that gate irrelevant stimuli before they consume valuable processing resources, and that effective therapies must restore these filtering functions. While computational psychiatry has long recognized that mental illness may reflect failures in predictive filtering, the specific neural substrate implementing this gating remains underspecified. Here the cerebellum emerges as a critical hub: neuroanatomically positioned to perform bottom-up sensory gating before cortical processing, housing more than half the brain’s neurons in an architecture ideally suited for distilling signal from noise and showing state-dependent disruption of cerebellar-cortical connectivity during symptom provocation in PTSD. Intriguingly, psychedelic drugs may act as recalibration triggers for these neural filters, acutely disrupting entrenched filtering architectures and reopening windows of plasticity through which maladaptive sensory weightings can be reset. This cerebellar filtering framework offers a neuroanatomically specified extension of predictive processing theory, generates falsifiable predictions, and suggests novel therapeutic targets for conditions that have resisted a half-century of receptor-focused drug development.

## 1 Introduction: the crisis in psychiatric drug development

The field of psychiatry faces a stark reality. Despite unprecedented investment in neuroscience research and drug development—with global spending exceeding $100 billion over the past two decades—there have been remarkably few genuine therapeutic innovations (1). The majority of psychiatric medications prescribed today trace their origins to serendipitous discoveries made between the 1950s and 1980s. Chlorpromazine for schizophrenia (1952), imipramine for depression (1957), and lithium for bipolar disorder (1970s) remain foundational treatments, with newer drugs primarily offering refinements in side effect profiles rather than fundamentally new mechanisms of action (2, 3).

This therapeutic stagnation has real consequences. Treatment-resistant depression affects approximately 30% of patients, with fewer than 40% achieving full remission even after multiple medication trials (4, 5). In schizophrenia, 60-70% of patients continue to experience persistent positive or negative symptoms despite antipsychotic treatment (6). For PTSD, available pharmacotherapies show limited efficacy, with only 20-30% of patients achieving remission with current FDA-approved medications (7). These statistics reveal not merely a need for better drugs, but a potential gap in our conceptual understanding of these disorders.

The traditional focus on neurotransmitter receptor modulation, e.g., the dopamine hypothesis of schizophrenia, the monoamine hypothesis of depression, the HPA axis dysregulation model of PTSD, has yielded important insights but appears incomplete. These frameworks conceptualize mental illness primarily as chemical imbalances and have guided the development of drugs that adjust synaptic concentrations of dopamine, serotonin, and norepinephrine. While these approaches provide symptomatic relief for many patients, they often fail to address the fundamental question - why does the brain misprocess information in the first place?

This treatise proposes a complementary framework: mental illness as a disorder of sensory and information filtering. Rather than focusing solely on which neurotransmitters are out of balance, this perspective examines how the brain gates, prioritizes, and processes the constant influx of sensory and internal information. When these filtering mechanisms fail, the organism becomes overwhelmed by noise that others effortlessly ignore, or conversely, becomes detached from salient stimuli. To appreciate why efficient information filtering would be critical for mental health, we must first consider the evolutionary pressures that shaped these mechanisms over hundreds of millions of years.

## 2 Evolutionary imperatives: Why efficient filtering matters

After hundreds of millions of years of evolution of life on Earth grown by the energy from the sun, there is a pyramid of energy transfer that starts with plants and ends with the apex predator. During our evolution we were part of the food chain, with senses fine-tuned for survival. The function of our sensory apparatus can be distilled to three evolutionary imperatives: find food, secure mates, and avoid predation.

### 2.1 Predation pressure and sensory filtering

Near-death experiences were likely common in ancestral environments. All animal life has developed sophisticated mechanisms to recognize natural threats and avoid predation. Indeed, many species—birds, opossums, rodents, fish, even sharks—can feign death (thanatosis) when threatened or in the clutches of a predator, a behavior that can facilitate escape (8). If these animals were traumatized by each near-death experience and unable to filter out the resulting negative memories, they would be unlikely to survive subsequent days.

This evolutionary logic suggests that animals in the wild must be exceptionally good at filtering, attending to sensory experiences and environmental situations that promote survival while rapidly suppressing those that do not. Humans, removed from direct predation pressure for millennia and living in evolutionarily novel environments, may have partially lost this adaptive capacity. Post-traumatic stress disorder serves as a stark illustration of what impaired filtering produces following a near-death or horrific experience — persistent hypervigilance, intrusive re-experiencing, and failure to habituate to non-threatening stimuli. Whether such filtering failures have been present across mammalian species and throughout human history, or represent a more recent phenomenon, does not alter the core argument: evolution strongly selected for rapid, efficient filtering of threat-associated memories precisely because organisms that could not achieve this would remain paralyzed by past experiences at the expense of present survival.

### 2.2 Two mechanisms of memory suppression: a critical distinction

Traumatic memories can be suppressed through two fundamentally different mechanisms and conflating them obscures therapeutic strategies.

#### 2.2.1 Extinction learning (top-down suppression)

This involves learning to inhibit the memory through a top-down process engaging the prefrontal cortex and hippocampus. The triggering stimulus is still fully perceived and processed, but cognitive control systems actively suppress the emotional and behavioral response (9–11). The traumatized victim hears the provocative noise, recognizes what it means, and chooses not to react. Critically, the original memory remains accessible and detailed—it has not been erased or filtered, merely inhibited.

#### 2.2.2 Sensory filtering (bottom-up gating)

This represents gating at the level of the brainstem and early sensory pathways. The triggering stimulus is filtered out before it reaches consciousness or memory systems. The victim does not fully process or even notice the trigger in the first place, and the original memory is thus less likely to be reactivated. This filtering occurs automatically, without requiring conscious effort or attention.

The distinction is crucial for several reasons. Extinction learning requires ongoing mental effort and attentional resources, making it vulnerable to stress, fatigue, and cognitive load. A patient who has learned to suppress their startle response through cognitive techniques may find this control slipping under conditions of exhaustion or acute stress. In contrast, sensory filtering operates automatically and economically, demanding minimal cortical resources. Once established, it operates continuously without conscious maintenance.

From an evolutionary perspective, nature would strongly favor the latter mechanism. Constantly using prefrontal resources to suppress responses to non-threatening stimuli would be metabolically expensive and would divert cognitive capacity from other survival-relevant tasks. It is far more adaptive to gate out irrelevant stimuli at the sensory level before they consume valuable processing resources. The evolutionary logic is clear: organisms that survived predation were those that could rapidly distinguish threatening from benign stimuli while conserving metabolic resources.

Of all the senses used to assess the environment for threat of predation, audition stands out. This is no accident. The same evolutionary logic that favors automatic, bottom-up sensory filtering applies most powerfully to our auditory system. To understand why auditory filtering is so critical—and why its failure proves so debilitating—we must first understand what makes hearing uniquely essential for survival.

## 3 The primacy of audition: sound as the sentinel sense

What makes audition uniquely important among our sensory modalities? The answer lies in the geometry of predation and survival. If an organism lived at the center of a sphere with threats potentially approaching from any direction -above, below, in front, behind, or from either side -only one sensory modality could rapidly and continuously monitor the entire space - hearing.

### 3.1 Omnidirectional threat detection

Vision, despite its remarkable acuity and information density, has a fundamental limitation - it is directional. Humans have approximately 180–200 degrees of visual field, with high-resolution central vision restricted to only a few degrees of foveal space (12). To see behind us, we must turn our heads. To see above or below, we must redirect our gaze. This creates blind spots, temporal windows during which threats could approach undetected. Olfaction and gustation provide chemical information about the environment but with significant temporal delays. Odor molecules must diffuse through air or dissolve in fluid before being detected. This delay can span seconds to minutes, far too slow for detecting an imminent predator. Additionally, olfactory information is heavily dependent on wind direction and environmental conditions, making it unreliable for 360-degree threat monitoring. Touch and proprioception operate only at the body surface or through direct mechanical contact. By the time a predator is within tactile range, escape options are severely limited. These modalities signal that a threat has already arrived rather than providing advance warning.

Sound, by contrast, propagates in all directions from its source and can be detected omnidirectionally by appropriately configured auditory systems. Most mammals, including humans, have two ears positioned on opposite sides of the head, enabling sophisticated sound localization through interaural time and intensity differences (13). This bilateral arrangement allows computation of sound source direction across the full 360-degree horizontal plane. Moreover, sound travels rapidly through air (approximately 343 meters per second at sea level) and propagates around obstacles that would block visual detection. A predator moving through underbrush, stepping on a twig, or displacing air through rapid movement creates acoustic signatures detectable well before visual contact. The rustling of leaves, the snap of a branch, the low-frequency rumble of approaching footsteps -these sounds provide early warning that vision cannot match.

### 3.2 Auditory vigilance during vulnerable states

Yet threats do not cease during sleep. Nocturnal predators specifically target sleeping prey precisely because they are vulnerable (14, 15). Evolution solved this problem by maintaining auditory processing during sleep. The auditory system remains operational throughout all sleep stages, continuously monitoring the acoustic environment (16). Sleep spindles and slow-wave activity during non-REM sleep enhance filtering of repetitive, non-salient sounds (the whir of a fan, distant traffic noise) while preserving responsiveness to novel or biologically significant sounds (a baby’s cry, one’s name, an alarm, or an unusual noise suggesting danger) (17, 18). This selective auditory gating during sleep represents an elegant evolutionary compromise. Complete sensory shutdown would maximize sleep’s restorative functions but would be catastrophically dangerous. Maintaining full auditory vigilance would prevent deep sleep and its associated benefits. The solution is sophisticated filtering: most sounds are gated out to protect sleep, but sounds with survival significance break through to trigger arousal (19, 20). This evolutionary logic explains why auditory gating deficits might be particularly debilitating. A person with impaired auditory filtering cannot achieve this compromise. Every sound—meaningful or meaningless—disrupts sleep or intrudes into waking consciousness. The constant barrage of auditory information that healthy brains effortlessly ignore becomes overwhelming.

### 3.3 Evolutionary pressure and auditory circuit elaboration

The unique importance of audition for survival shaped the evolution of auditory processing circuits. The mammalian auditory system exhibits remarkable anatomical and functional elaboration compared to other sensory pathways. The cochlea contains approximately 16,000-20,000 hair cells in humans, each tuned to specific frequency ranges, providing exquisite frequency discrimination. The cochlear nucleus, the first central auditory relay, contains multiple specialized cell types and performs sophisticated processing including temporal pattern recognition and binaural comparison.

The ascending auditory pathway includes multiple parallel streams with specialized functions, precise temporal encoding for sound localization, intensity coding for threat assessment, and frequency analysis for sound identification. Critically, auditory information reaches the cerebellum very early in this pathway, with direct projections from the cochlear nucleus to the cerebellar vermis and interposed nuclei (21, 22). This early cerebellar access positions the cerebellum to gate auditory information before it reaches cortical areas - exactly what an efficient filtering system requires.

The startle response, one of the most evolutionarily conserved defensive behaviors, is primarily triggered by sudden loud sounds rather than other sensory modalities. Its latency is remarkably short, typically 10–20 milliseconds from sound onset to initial muscle activation, far faster than cortically mediated reactions (23). This subcortical speed reflects evolutionary prioritization: rapid auditory threat detection and response can mean the difference between survival and death.

### 3.4 Implications for psychiatric filtering deficits

If audition occupies a privileged position in threat detection and vigilance, then auditory filtering deficits should be particularly evident in psychiatric conditions characterized by hypervigilance and threat sensitivity. This prediction is strongly supported by clinical evidence. Prepulse inhibition and P50 suppression - the gold-standard measures of sensory gating - are both auditory paradigms. While (PPI) can be tested with other modalities (tactile, visual), the acoustic version is most commonly used and shows the most robust clinical differentiation (24). Patients with schizophrenia and PTSD show consistent deficits specifically in auditory gating (25–27). Moreover, many psychiatric symptoms have auditory components. Auditory hallucinations in schizophrenia are more common than visual hallucinations (28). Hyperacusis (heightened sound sensitivity) is reported in PTSD, autism, and anxiety disorders (27, 29). Misophonia, strong negative reactions to specific sounds like chewing or breathing, has been linked to altered sensory gating (30). These auditory symptoms may reflect the consequence of failing to filter out irrelevant sounds in a modality that evolution designated as critical. This primacy of audition also suggests therapeutic strategies. If auditory filtering is particularly important and particularly vulnerable to disruption, then interventions specifically targeting auditory gating might be especially effective. Sound-based therapies, auditory training paradigms, or drugs that enhance auditory filtering specifically could complement broader approaches.

### 3.5 Summary: sound as the sentinel

Throughout evolutionary history, sound has served as the sentinel sense—the modality that monitors for threats when others cannot. Its omnidirectional propagation, rapid transmission, and continuous operation during sleep make it uniquely suited for vigilance. The elaborate neural circuits devoted to auditory processing and gating reflect millions of years of selection pressure favoring organisms that could efficiently filter acoustic noise while remaining responsive to threats. When these filtering mechanisms fail, as they do in multiple psychiatric conditions, the organism loses its sentinel. Every sound becomes potentially threatening, every noise demands attention, and the acoustic environment transforms from background to foreground. The patient cannot sleep deeply because benign sounds trigger arousal. They cannot concentrate because irrelevant conversations intrude. They cannot relax because the soundscape remains threateningly salient.

Understanding the primacy of audition clarifies why sensory gating, particularly auditory gating, is so central to mental health. It also suggests that successfully treating psychiatric disorders may require restoring the brain’s oldest and most fundamental defense mechanism: the ability to distinguish between sounds that matter and sounds that don’t, between signals warning of danger and noise that can be safely ignored. Having established why auditory filtering is evolutionarily critical and uniquely vulnerable to disruption, we must now examine the neurobiological mechanisms that implement this filtering. How does the nervous system actually distinguish signal from noise, and what objective measures can we use to detect when these mechanisms fail?

## 4 Sensory gating: neurobiological mechanisms and clinical evidence

Our brains are ceaselessly inundated by sensory inputs—sights, sounds, smells, touches, proprioceptive feedback—yet only a tiny fraction reach conscious awareness. This selective gating is not a luxury; it is a necessity for functioning in any complex environment. Without it, we would be paralyzed by information overload, unable to distinguish signal from noise, relevant from irrelevant.

### 4.1 Prepulse inhibition and P50 suppression

Neuroscience has long studied sensory gating phenomena using standardized paradigms. Two of the most well-established are: *Prepulse Inhibition (PPI)* When a weak pre-sound (the prepulse) is presented 30–500 milliseconds before a loud startling noise, it suppresses the magnitude of the startle response. This phenomenon demonstrates that the nervous system can use predictive information to gate auditory incoming stimuli, reducing responses to expected or redundant inputs, and *P50 Auditory Evoked Potential Suppression* When two identical clicks are presented 500 milliseconds apart, the brain response (measured via EEG) to the second click is substantially reduced compared to the first. Healthy individuals show 50-80% suppression of the P50 wave to the second click, indicating efficient gating of repetitive auditory information. In healthy brains, these tests demonstrate the nervous system’s remarkable ability to filter out repetitive, irrelevant noise. The first click or the first loud noise is novel and potentially important; the second, occurring soon after, likely represents redundant information and can be safely attenuated.

### 4.2 Gating deficits across psychiatric disorders

Strikingly, many psychiatric disorders show consistent deficits in these gating measures as noted above. Patients with schizophrenia show reduced PPI (typically 30-40% reduction compared to healthy controls) and diminished P50 suppression (often less than 20% suppression of the second click response) (31, 32). Similar deficits have been documented in bipolar disorder, obsessive-compulsive disorder, autism spectrum disorder, and post-traumatic stress disorder (24, 33). Patients with these conditions cannot efficiently filter out repetitive auditory stimuli. They are, in a neurophysiological sense, flooded by noise that others effortlessly ignore. This observation reinforces the idea that a core feature of many mental illnesses is the failure of the brain’s sensory filters. The downstream consequences are severe: inability to concentrate, intrusive thoughts, hallucinations, hypervigilance, and emotional dysregulation can all be understood, at least in part, as secondary effects of failed sensory gating.

### 4.3 Neural circuits of sensory auditory gating

Where and how does this filtering occur? The answer involves multiple levels of the neuroaxis, from brainstem to cortex.

### 4.4 Thalamocortical circuits

At one level, circuits in the thalamus act as relays that can amplify or dampen signals before they reach the cortex. The thalamic reticular nucleus, a shell of GABAergic neurons surrounding the thalamus, serves as a critical gating structure. A thalamocortical loop involving the sensory cortices, prefrontal cortex, basal ganglia, and reticular nucleus regulates attention and gating of sensory input during wakefulness (34). This circuit has been extended to include the hippocampus (for contextual memory) and amygdala (for emotional salience). Recent work has demonstrated that the prefrontal cortex regulates sensory filtering through a basal ganglia-to-thalamus pathway, allowing top-down control of which sensory streams reach conscious awareness. Lesions or dysfunction in any component of this circuit can produce gating deficits.

### 4.5 Brainstem and midbrain pathways

Auditory gating has a particularly well-defined neural circuitry beginning in the brainstem cochlear nuclei (21, 22). The cochlear nucleus projects to the nearby superior olivary complex and, critically, to the cerebellum. From there, the pathway continues to the inferior colliculus of the midbrain, then to the medial geniculate nucleus in the thalamus, and finally to the auditory cortex and the corticothalamic feedback loop. The PPI of the startle reflex can be observed at the brainstem level, in the nucleus reticularis pontis caudalis, without requiring cortical decision-making (35). This demonstrates that filtering can occur very early in sensory processing, before information ever reaches conscious awareness.

### 4.6 Sleep and continuous filtering

Even during sleep, when consciousness is minimal or absent, the brain continues to filter sensory information. As noted above, sleep spindles and slow waves during non-REM sleep actively enhance sensory gating, preventing most external stimuli from disrupting sleep. However, auditory processing remains partially active even during deep sleep, with recent evidence showing that auditory processing reaches the cortex during sleep spindles (18), maintaining vigilance for salient sounds (like a baby’s cry or one’s own name) that might signal danger or need. The continuous operation of these filtering systems, even during sleep, underscores their fundamental importance and raises a critical question: what is the metabolic cost of this constant vigilance, and how might the brain minimize that cost?

## 5 Energy economics of neural filtering

Before examining how filtering is regulated, we must understand why bottom-up, automatic filtering is not merely one option but likely represents the brain’s primary strategy. The answer lies in the fundamental economics of neural computation.

### 5.1 Brain and metabolism

The brain consumes approximately 20% of the body’s energy budget despite comprising only 2% of body weight. Roughly 75% of this energy supports neural signaling, with synaptic transmission - not computation - accounting for the largest portion (44-47%) of brain energy consumption (36). Each synaptic event requires ATP to restore ion gradients, synthesize and recycle neurotransmitters, and maintain cellular homeostasis. Interestingly, the brain maintains remarkably high energy consumption regardless of activity level, awake or sleeping (37), suggesting that sensory filtering systems operate continuously at high metabolic cost. Given that evolution strongly optimizes for metabolic efficiency, particularly in the energetically expensive brain, it makes adaptive sense that the nervous system would offload as much gating as possible to lower, more energy-efficient levels before engaging expensive cortical resources.

Consider the alternatives. If every sound, visual stimulus, tactile sensation, and proprioceptive signal had to reach the cortex before being evaluated for relevance, the metabolic burden would be prohibitive. Cortical processing involves extensive recurrent connectivity, working memory maintenance, and integration with executive functions, all energetically expensive operations. By gating irrelevant information at the brainstem or thalamic level, the brain can operate far more efficiently. This economic argument suggests that bottom-up filtering i.e., “noise cancellation” that happens automatically as raw sensory data flows inward, is not just one option among many, but likely represents the primary, evolutionarily conserved strategy. Top-down, cortically-mediated filtering exists and is important, but it should be viewed as a supplementary system for handling novel or ambiguous situations that cannot be resolved by automatic gating.

### 5.2 Therapeutic implications of the filtering framework

If mental illnesses fundamentally involve a breakdown of these filters, then reinstating efficient bottom-up gating could be more effective and enduring than attempting to enforce top-down cognitive control. A person can learn through therapy to ignore hallucinations or intrusive memories, a top-down approach, but this strategy is metabolically expensive and prone to failure under stress. The patient must continuously expend cognitive resources to suppress responses that their filtering system should be handling automatically. By contrast, if a treatment can eliminate the false signal altogether, if it can make the brain inherently unresponsive to irrelevant noise or fearful cues, the person would not need to constantly fight those symptoms. The system would self-correct. Achieving this ambitious goal requires understanding and targeting the neural circuits that perform automatic filtering. The cerebellum, with its massive computational capacity and strategic position, provides this energetically efficient solution.

## 6 The cerebellum: from motor coordinator to master filter

Where in the brain might automatic, bottom-up filtering reside? The answer, increasingly supported by modern neuroscience, points to a structure traditionally associated with motor control - the cerebellum. This may seem counterintuitive with the cerebellum as a cognitive and emotional regulator, but emerging evidence has fundamentally changed our understanding of cerebellar function.

### 6.1 Cerebellar anatomy and connectivity

The cerebellum contains more neurons than the rest of the brain combined. The human cerebellum contains approximately 69 billion neurons, representing about 80% of the brain’s total 86 billion neurons, while the cerebral cortex contains only about 16 billion neurons (approximately 19% of total brain neurons) (38, 39). This massive neuronal population is predominantly composed of tiny granule cells packed into a dense layer beneath the cerebellar cortex. Despite housing more than half the brain’s neurons, the cerebellum communicates with the rest of the brain via only three pairs of deep cerebellar nuclei: the dentate, interposed, and fastigial nuclei.

This organization, with massive parallel processing funneled through narrow output channels suggests that the cerebellum functions as a computational hub that distills enormous amounts of information into refined control signals. The structure does not initiate thought or action like the cortex does; instead, it modulates and fine-tunes neural activity across multiple domains. This is precisely the architecture evolution would construct for a noise filter. The cerebellum receives diverse inputs, performs extensive processing to distinguish signal from noise, and outputs cleaned, filtered information to the rest of the brain. Importantly, the cerebellum is densely interconnected with cognitive, emotional, and sensory centers (40). Cerebello-thalamo-cortical circuits link the cerebellum to prefrontal cortex, posterior parietal cortex, and temporal lobe structures. The cerebellum receives inputs from virtually every sensory modality and sends outputs that modulate activity in limbic structures including the amygdala and hippocampus. These connections position the cerebellum to influence not only motor coordination but also attention, emotion, memory, and sensory processing. Indeed, over the past three decades, evidence has accumulated demonstrating the cerebellum’s involvement in non-motor functions. Cerebellar lesions in humans produce a characteristic syndrome - cerebellar cognitive-affective syndrome (CCAS) - featuring executive dysfunction, spatial cognition deficits, linguistic difficulties, and affective dysregulation (41). These patients may exhibit flattened affect, disinhibited or inappropriate behavior, and difficulty with abstract reasoning, despite having intact motor function in some cases. Neuroimaging studies consistently demonstrate cerebellar activation during cognitive tasks including working memory, language processing, attention, and emotional processing (42, 43).

### 6.2 Cerebellar peduncular architecture: input-output pathways for filtering

The anatomical connections between the cerebellum and the rest of the brain are mediated by three pairs of cerebellar peduncles—large white matter fiber tracts that serve as the physical substrate for filtering the flow of information.

### 6.3 The inferior cerebellar peduncle: sensory input and prediction errors

The ICP represents the primary input pathway carrying sensory information to the cerebellum from brainstem and spinal sources (44). It carries two critical types of information: 1) Climbing fibers from the inferior olivary complex: These constitute the most important input for the filtering framework. Inferior olivary neurons detect unexpected sensory events—mismatches between prediction and reality—and signal these prediction errors to the cerebellum via climbing fibers (45). Each climbing fiber makes hundreds of excitatory synapses onto a single Purkinje cell, creating a powerful teaching signal that drives cerebellar learning and filter recalibration. 2) Spinocerebellar tracts: Carry proprioceptive information from the body, providing continuous feedback about limb position, muscle tension, and movement. This proprioceptive stream is essential for filtering motor noise and predicting the sensory consequences of movement.

### 6.4 The middle cerebellar peduncle: cortical context input

The MCP is the largest peduncle and is purely afferent, carrying cortical information to the cerebellum via the cortico-ponto-cerebellar pathway (46). Widespread cortical regions (prefrontal, parietal, temporal, sensory, limbic cortices) send projections through pontine nuclei to reach the cerebellar cortex as mossy fibers. Projections from the prefrontal cortex convey information about current goals, task context, emotional state, and threat assessment (47). This top-down contextual information allows the cerebellum to adjust its filtering thresholds appropriately. In a safe therapeutic environment, prefrontal input via the MCP should signal the cerebellum to reduce threat-related filtering. Projections from limbic and sensory cortices provide high-level processed sensory information and emotional/motivational content, enabling the cerebellum to modulate filtering based on cortical interpretation and affective state (48).

### 6.5 The superior cerebellar peduncle: filtered output to cortex

The SCP is the primary efferent pathway of the cerebellum, carrying filtered signals from the deep cerebellar nuclei to the thalamus and then to cortex (46). The SCP decussates in the ventral midbrain, with each cerebellar hemisphere affecting the contralateral cerebral cortex. The SCP originates from three deep cerebellar bilateral nuclei each with distinct functions: 1) The dentate nuclei project to the ventrolateral/mediodorsal thalamus and prefrontal/premotor cortex (49). Transmits cognitively-filtered information back to prefrontal cortex, enabling executive control, working memory, and emotional regulation; 2) The interposed nuclei project to the ventrolateral thalamus and motor cortex and carries movement-related filtering outputs, ensuring smooth motor control (50); 3) The fastigial nuclei project to the reticular formation, vestibular nuclei and brainstem structures controlling arousal, balance, and autonomic function (51). The state-dependent cerebello-thalamo-cortical hypoconnectivity observed by Kearney et al. in PTSD patients during traumatic memory retrieval most likely reflects disrupted SCP function. During trauma recall, filtered safety signals from the cerebellum fail to reach cortex via the SCP, allowing the unfiltered trauma circuit to dominate conscious experience. In depression, reduced SCP connectivity from the dentate nucleus to prefrontal cortex may prevent filtered positive information from reaching consciousness, trapping the patient in negative cognitive patterns.

### 6.6 Summary: the cerebellar peduncles as a filtering circuit

The three cerebellar peduncles form an integrated filtering circuit with complementary roles. The cerebellum receives bottom-up sensory inputs and prediction errors (ICP: inferior olive, spinocerebellar tracts) combined with top-down cortical context and goals (MCP: cortico-ponto-cerebellar pathway). Within the cerebellar cortex, these inputs converge on Purkinje cells, which integrate climbing fiber teaching signals with mossy fiber contextual information. The massive computational capacity (69 billion neurons) enables sophisticated filtering decisions: signal versus noise, expected versus unexpected, threat versus safety. These processed, filtered decisions are transmitted back to cortex (via SCP to thalamus) and brainstem (via fastigial nucleus to vestibular/reticular nuclei).

### 6.7 The inferior olive-cerebellar circuit: the “great gate”

A critical insight into cerebellar filtering comes from understanding the relationship between the inferior olivary complex and the cerebellum, a relationship Anna Devor has termed the “Great Gate” (45). The inferior olive, located in the medulla, contains neurons that are exquisitely tuned to novel sensory events across all modalities. These neurons exhibit a peculiar property: they fire when a stimulus is unexpected or when there is a mismatch between prediction and reality. Olivary neurons send climbing fibers directly into the cerebellum, each climbing fiber making hundreds of excitatory synapses onto a single Purkinje cell. This climbing fiber input essentially alerts the cerebellum to prediction errors i.e., moments when sensory reality deviates from expectation. In response, the cerebellum can send inhibitory feedback to shut down the inferior olive’s activity, closing the gate. Through this reciprocal loop, the cerebellum functions as both a prediction machine and a comparator (52, 53). When a sensory input aligns with expectation, the cerebellum helps cancel it out as noise; when it deviates, the cerebellum lets it through or even amplifies it as signal requiring attention. This mechanism aligns perfectly with the framework of predictive coding, wherein the brain constantly generates predictions about incoming sensory data and devotes processing resources primarily to prediction errors rather than redundant, expected information.

The climbing fiber system operates with precise temporal dynamics. Climbing fiber activation produces a complex spike in Purkinje cells, a distinctive electrical event that triggers synaptic plasticity in the parallel fiber inputs (the other major input to Purkinje cells, conveying mossy fiber information from throughout the brain). This plasticity mechanism allows the cerebellum to continuously update its internal models based on experience, refining its predictions and, consequently, its filtering accuracy over time (54, 55). Importantly, this filtering mechanism is not limited to motor or simple sensory information. The cerebellum receives inputs from limbic and association cortices, and the same predictive cancellation algorithm could theoretically apply to thoughts and emotions. Ruminative or intrusive thoughts could be conceptualized as “internal noise”, repetitive, non-novel cognitive patterns, that an intact cerebellar system might help suppress. When cerebellar function is disrupted, both external sensory noise and internal cognitive noise might proliferate.

## 7 Neurotransmitter systems as filtering regulators

The consistent involvement of cerebellar circuits across psychiatric disorders raises a fundamental question: what regulates these filtering systems? While the anatomical pathways (peduncles) and computational architecture (Purkinje cells, climbing/mossy fibers) provide the substrate for filtering, the dynamic adjustment of filtering thresholds requires neuromodulation. Three monoaminergic systems serotonergic, dopaminergic, and noradrenergic serve as the chemical regulators that tune cerebellar filtering to match environmental demands and internal states.

### 7.1 Serotonin: global gating threshold modulation

Serotonergic (5-HT) projections from raphe nuclei densely innervate the cerebellum (Purkinje cells, granule cells, deep nuclei) and inferior olive (56–58). 5-HT modulates Purkinje cell excitability and could be effectively adjusting the gain of cerebellar filtering. For example, low 5-HT levels would be expected to reduce Purkinje cell inhibition leading to less efficient gating of mossy/climbing fiber inputs (59, 60). The consequences would be more unfiltered sensory and cognitive noise reaching cortex. 5-HT2A receptors (the target of psychedelics) are highly expressed on Purkinje cells (61, 62) and inferior olivary neurons (63, 64). This treatise proposes psychedelic activation of 5-HT2A receptors disrupts the inferior olive-cerebellar ‘Great Gate’ (45), allowing the flow of massive unfiltered information characteristic of the acute psychedelic state. This mechanism is consistent with observed sensorimotor gating impairments (65) and whole-brain desynchronization (66) following administration of psychedelics. Serotonin-specific reuptake inhibitors may work not by directly improving mood but by restoring serotonergic modulation of cerebellar filtering, allowing more efficient suppression of ruminative thoughts (internal cognitive noise).

### 7.2 Dopamine: salience weighting control

Dopaminergic (DA) input to the cerebellum is indirect and comes via closed loops with the basal ganglia (67–70). DA could function by modulating salience filtering, marking which stimuli are rewarding or threatening (71). In schizophrenia, excessive striatal DA may override cerebellar filters by inappropriately marking internal events (thoughts, perceptions) as externally salient, contributing to hallucinations and delusions (72, 73). Antipsychotics reduce DA signaling, potentially restoring appropriate salience filtering by allowing cerebellar gates to function without being overridden by aberrant striatal signals (74).

### 7.3 Norepinephrine: arousal-dependent filtering sensitivity

Noradrenergic (NE) projections to the cerebellum come predominately from the locus coeruleus (75). In all probability, the noradrenergic projections to cerebellum modulate filtering as a function of arousal state (76). For example, high NE activity would lower filtering thresholds (hypervigilance), appropriate during acute threat. However, in cases of PTSD and anxiety, chronically elevated NE maintains hypervigilant filtering even in safe contexts (77). Interestingly, Prazosin (alpha-1 antagonist) for PTSD nightmares may work by reducing NE drive on cerebellar circuits during sleep, allowing normal sensory gating to resume (78).

Thus, neurotransmitters are not merely mood modulators but may regulate filtering operating at specific nodes in the cerebellar circuit. 5-HT adjusts Purkinje cell gain and olivary excitability, setting global filtering thresholds. DA modulates striato-cerebellar loops that determine salience weighting. NE dynamically adjusts filtering as a function of arousal state. Psychiatric medications may work by restoring appropriate neurotransmitter-mediated filtering parameters. However, this framework suggests that optimal treatment may require not just normalizing neurotransmitter levels but ensuring those neurotransmitters can properly modulate cerebellar circuits—a hypothesis testable via imaging studies examining drug-induced changes in cerebellar-thalamo-cortical connectivity.

### 7.4 Summary: neurotransmitters as dynamic filter controllers

Thus, neurotransmitters are not merely mood modulators but may regulate filtering operating at specific nodes in the cerebellar circuit. 5-HT adjusts Purkinje cell gain and olivary excitability, setting global filtering thresholds. DA modulates striato-cerebellar loops that determine salience weighting. NE dynamically adjusts filtering as a function of arousal state.

## 8 Three types of filtering dysfunction and their disorder-specific manifestations

A crucial question remains: if filtering failure is a common mechanism across psychiatric disorders, why do different conditions produce such distinct symptom profiles? How does the same PPI deficit lead to auditory hallucinations in one patient and hypervigilance in another? The answer lies in recognizing that “filtering” is not monolithic but encompasses at least three distinct types of information processing, each mediated by partially separable neural circuits and each vulnerable to disorder-specific disruption.

### 8.1 Type 1: external sensory filtering (bottom-up gating)

This represents the failure to gate repetitive or irrelevant sensory inputs at brainstem/cerebellar and thalamic levels before they reach cortical processing. The filtering occurs automatically, without requiring conscious effort, through circuits involving the cochlear nucleus, inferior colliculus, thalamic reticular nucleus, and their reciprocal connections with the cerebellum (21, 22). When this system fails, the person becomes overwhelmed by sensory noise that healthy individuals effortlessly suppress. The primary examples are schizophrenia and PTSD.

#### 8.1.1 Schizophrenia: external sensory filter collapse

Schizophrenia, perhaps the quintessential disorder of sensory overload and information processing dysfunction, has been consistently associated with cerebellar abnormalities. Numerous neuroimaging studies confirm structural and functional cerebellar abnormalities in schizophrenia (79–83). These changes include reduced cerebellar gray matter volume, particularly in posterior lobules; altered cerebello-thalamo-cortical connectivity, with reduced functional coupling between cerebellum and prefrontal cortex; abnormal cerebellar activation patterns during cognitive tasks; and dysfunction of the cerebellar dentate nucleus showing reduced connectivity with frontoparietal networks. These abnormalities appear early in the disease course, present even in first-episode, drug-naive patients and in individuals at ultra-high risk for psychosis, suggesting they represent core pathophysiology rather than secondary effects of chronic illness or medication.

In schizophrenia, the filtering deficit is predominantly external-sensory: the cerebellum’s inability to gate auditory input via the inferior olivary-cerebellar-thalamic pathway allows internally-generated auditory patterns (normally suppressed as noise) to reach consciousness as perceived external voices (81, 84). This is why auditory hallucinations are more common than visual (60–80% vs. 27%) (85), and why PPI deficits specifically in the auditory modality are the most robust biomarker (24, 86). The uncontrollable nature of voices correlates directly with the severity of gating deficits—patients who feel they have no control over their hallucinations show the most marked PPI impairment (86).

The filtering hypothesis offers a parsimonious explanation for the full range of schizophrenia symptoms. If cerebellar-mediated filtering fails, the cortex becomes inundated with unfiltered sensory and internal information. Hallucinations represent internally generated neural activity that would normally be suppressed by predictive cancellation but instead reaches consciousness as perceived external stimuli. Delusions might emerge from the brain’s attempt to make sense of a chaotic, unfiltered stream of perceptions. Cognitive fragmentation and attention deficits follow naturally from the inability to separate relevant from irrelevant information.

#### 8.1.2 Post-traumatic stress disorder: state-dependent filter failure

While schizophrenia represents a relatively constant failure of external sensory filtering, PTSD demonstrates an equally compelling but mechanistically distinct pattern: filtering failure that emerges specifically when confronted with trauma-relevant information.

Recent evidence provides particularly compelling support for the cerebellar filtering hypothesis in PTSD. Kearney and colleagues used whole-brain functional connectivity analysis to examine brain networks during traumatic memory retrieval versus neutral memory retrieval in PTSD patients (87). They found striking patterns of cerebellar hypoconnectivity with thalamic, frontal, and basal ganglia regions—but critically, this connectivity breakdown occurred specifically during traumatic memory retrieval, not during neutral memory processing. This state-dependent disruption is theoretically crucial: it demonstrates that the filtering dysfunction is not a general deficit but rather a failure that emerges precisely when it is most needed, during the processing of threatening or overwhelming information. The cerebellum appears unable to properly gate trauma-related inputs, allowing them to flood the cortex and trigger the full constellation of PTSD symptoms—hyperarousal, intrusive memories, flashbacks, and hypervigilance. Moreover, the magnitude of these cerebellar connectivity alterations correlated with clinical symptom severity, providing direct evidence that cerebellar filtering capacity relates to real-world functional impairment.

In PTSD, external sensory filtering failure manifests as hypervigilance and exaggerated startle responses to objectively benign environmental sounds (27). The brain cannot suppress trauma-associated acoustic cues—sudden noises, specific voices, environmental sounds similar to those present during the traumatic event—leading to persistent activation of threat circuits even in safe contexts.

This finding also validates the distinction between filtering and extinction learning introduced in **Section 2**. Traditional exposure therapy for PTSD works through extinction learning, a top-down, cortically-mediated process requiring intact prefrontal-hippocampal-amygdala circuits. While effective for many patients, extinction-based approaches require ongoing cognitive effort and remain vulnerable to renewal, reinstatement, and spontaneous recovery of the fear response. The cerebellar filtering deficit suggests a complementary target: if the cerebellum’s ability to gate trauma-related inputs at the sensory level could be restored, traumatic memories might lose their capacity to intrude into consciousness in the first place.

### 8.2 Type 2: internal cognitive/affective filtering

This represents the failure to suppress ruminative thoughts that would normally be gated as non-productive mental activity. Unlike external sensory filtering, which operates at subcortical levels, internal filtering involves cerebellar modulation of prefrontal-limbic circuits including the default mode network, ventromedial prefrontal cortex, and amygdala (88). When this system fails, maladaptive thought patterns that should be suppressed as “internal noise” instead dominate consciousness. The primary examples are major depressive disorder (MDD), obsessive-compulsive disorder, and generalized anxiety disorder.

#### 8.2.1 Major depressive disorder: internal filter failure

In contrast to schizophrenia, MDD shows a fundamentally different pattern: relatively intact external sensory gating but profound failure of internal cognitive/affective filtering. Critically, patients with depressive disorder show normal prepulse inhibition, with little difference from healthy controls, helping to differentiate them from schizophrenia patients who show robust PPI deficits (89–91). This preserved external gating indicates that the brainstem-cerebellar-thalamic sensory filter remains functional.

However, the dysfunction involves cerebellar modulation of prefrontal-limbic circuits, as negative self-referential thoughts and anhedonic responses that should be suppressed as non-adaptive mental noise instead become dominant. Neuroimaging studies consistently demonstrate reduced functional connectivity between the cerebellum and ventromedial prefrontal cortex in depression, with the severity of this disconnection correlating with depression symptoms (88). This cerebellar-prefrontal disconnection is most evident during negative emotional processing, where the cerebellum fails to gate repetitive internal cognitive patterns—rumination, negative self-referential thought, and catastrophic prediction—mental activity that should be filtered as redundant and maladaptive. Clinical manifestations include persistent rumination resistant to attentional redirection, anhedonia reflecting failed positive stimulus processing, intrusive negative cognition, and impaired cognitive flexibility. These findings indicate that depression treatment must target cerebellar-prefrontal filtering circuits rather than external sensory gating mechanisms, which remain functionally intact.

### 8.3 Type 3: Contextual/salience filtering

This represents the failure to appropriately weight the importance of stimuli based on context—determining what deserves attention versus what can be ignored. This system involves cerebello-striatal-prefrontal circuits mediating attentional control and salience attribution (34, 82). When this filtering fails, the organism either attends to irrelevant stimuli or fails to respond to important ones, producing disorders of attention and social cognition. Primary examples include autism spectrum disorder and attention-deficit/hyperactivity disorder.

#### 8.3.1 Autism spectrum disorder and ADHD: contextual filter dysfunction

In autism spectrum disorder, contextual filtering manifests as an inability to filter socially-relevant from socially-irrelevant information. Individuals with ASD may attend intensely to non-social stimuli (textures, patterns, mechanical objects) while failing to automatically orient to social cues (facial expressions, vocal prosody, gestures) that typically capture attention (92). This is not simply a preference but a filtering failure: the salience weighting system assigns inappropriate priorities. Sensory over-responsivity in autism—including hyperacusis (heightened sound sensitivity)—reflects this contextual filtering deficit (29). The brain fails to adjust filtering thresholds based on social context, treating all stimuli with equal importance regardless of their relevance to ongoing social interaction.

### 8.4 Summary: a taxonomy of filtering dysfunction

These three filtering types provide a taxonomy for understanding psychiatric heterogeneity within a unified framework. Importantly, the disorders are not mutually exclusive—many patients show deficits across multiple filtering types. Comorbidity between PTSD and depression, or between ADHD and anxiety, may reflect overlapping filtering dysfunctions affecting both external sensory and internal cognitive systems. The consistency of cerebellar involvement across diagnostically diverse conditions supports a transdiagnostic role for cerebellar filtering, with different disorders reflecting different patterns of dysfunction within the same underlying architecture.

This classification also has direct therapeutic implications. Therapies effective for one filtering type may be ineffective or even counterproductive for another. Traditional exposure therapy for PTSD works through top-down extinction (Type 1 filtering enhancement) but may be less effective for depression (Type 2 filtering failure). Psychedelic-assisted therapy may reset filters across multiple types, explaining its transdiagnostic potential. By distinguishing these filtering types and their associated neural circuits, the framework escapes the overgeneralization critique while retaining its core insight: psychiatric symptoms reflect failures of the brain’s evolved information processing architecture. The question is not whether filtering fails, but which filter, in which circuit, producing which symptoms.

With this taxonomy established, we can now examine a class of drugs that may offer an unprecedented ability to reset these mechanisms across multiple filtering types: classical psychedelics.

## 9 Psychedelics: resetting the brain’s filters

Classical psychedelic drugs—lysergic acid diethylamide (LSD), psilocybin, mescaline, and N,N-dimethyltryptamine (DMT)—are known for dramatically altering perception (93, 94). Users often report that senses “mix” or intensify (synesthesia), that the boundary between self and environment blurs (ego dissolution), or that ordinary inputs take on profound new significance. At first glance, this might seem the opposite of what we want in treating disorders of sensory overload. How could drugs that cause hallucinations help someone who already hears voices or relives traumatic memories? The answer lies in distinguishing acute effects from lasting therapeutic changes. Psychedelics transiently perturb the brain’s networks during the acute experience, but they also initiate a profound period of neural plasticity—a rewiring window during which networks can reform in healthier patterns (95). Rather than suppressing symptoms, psychedelics may reset the fundamental parameters of sensory filtering, allowing maladaptive filters to be recalibrated.

### 9.1 LSD, psilocybin, mescaline and cerebellar hyperconnectivity

Recent neuroimaging studies in my lab revealed something striking; psychedelics strongly affect the cerebellum and its connectivity with the rest of the brain. In fully awake rats given LSD during the MRI scanning session, we reported an increase in functional connectivity across many networks (96). The most unexpected finding was a dramatic hyperconnectivity between the cerebellum, including the deep cerebellar nuclei, and numerous other brain regions under LSD. In a subsequent study, we also reported psilocybin produced a similar effect – heightened synchronization between the cerebellum and cortical areas, especially at higher doses (97). Mescaline also causes cerebellar hyperconnectivity (Cavallaro et al., 2025 under review). This common effect of classical psychedelics is noteworthy because such widespread cerebellar connectivity is not typically seen. It suggests that psychedelics temporarily open the floodgates of the cerebellum. Why would this be therapeutic? Perhaps by breaking down entrenched networks and then re-establishing connections, the cerebellum might recalibrate its filtering thresholds.

The acute hyperconnectivity we observed during psychedelic sessions reveals the first phase of filter reset: a controlled collapse of existing filtering architectures. But acute disruption alone does not explain therapeutic benefit. To understand the healing mechanism, we must examine what psychedelics do to sensory salience itself.

### 9.2 Mescaline and altered sensory perception: recalibrating innate salience

Our most recent study using mescaline added another critical dimension. During the scanning session we challenged the rats with a specific sensory stimulus to determine whether perception in that modality was altered. The stimulus was benzaldehyde, the scent of almonds, which is particularly interesting from an evolutionary perspective. Rats are innately attracted to benzaldehyde. This is an evolutionarily conserved response: almonds have high caloric density, can be stored for extended periods, and their recognition is so fundamental that mammals (including humans) possess specific olfactory receptors coded to detect benzaldehyde (98, 99). When odor-naive rats are presented with almond scent during scanning, they show robust activation of neural circuitry associated with motivation and reward—far stronger than responses to other novel odorants. Remarkably, mescaline-treated rats no longer showed this characteristic activation pattern. The drug made a previously highly salient, evolutionarily meaningful stimulus become less attention-grabbing. The olfactory and reward circuits that normally light up in response to benzaldehyde showed markedly attenuated activation under mescaline.

### 9.3 Therapeutic implications

This hints that psychedelics can reset what the brain deems important or salient—a fundamental recalibration of sensory filtering priorities. This finding has direct therapeutic implications. If a psychedelic can reduce the salience of an innate, hardwired attractive stimulus, might it also reduce the salience of learned, maladaptive associations? Could a trauma-associated trigger, which has become pathologically salient through fear conditioning, lose its power to capture attention and provoke distress?

The mescaline-benzaldehyde finding demonstrates that psychedelics can fundamentally recalibrate what the brain deems important—reducing the salience of even evolutionarily hardwired stimuli. But this raises a paradox: if psychedelics disrupt filtering, how could they help conditions already characterized by filtering failure? The answer becomes clear when we examine their frequency-specific effects.

### 9.4 Psychedelics and prepulse inhibition: context-dependent effects

We also tested mescaline’s effects on PPI using acoustic startle paradigms. Rats were exposed to a 70 dB prepulse followed by a 120 dB startle stimulus, at three different frequencies: 4 kHz (low), 12 kHz (middle), and 20 kHz (high). The results were unexpected and theoretically important. Mescaline significantly enhanced gating (stronger PPI) at the low (4 kHz) and high (20 kHz) frequencies, but impaired gating at the middle frequency (12 kHz). The complexity of this finding serves as a caution. Psychedelic effects on filtering are nuanced and frequency, or context-dependent. Simple narratives about psychedelics “opening” or “enhancing” sensory gating may miss important specificity. Different patients, with different symptom profiles, might benefit from different psychedelic compounds or doses that produce optimal recalibration for their particular filtering deficits. The frequency-specific effects reveal that psychedelics don’t uniformly impair or enhance filtering, but rather produce nuanced, context-dependent modulation. This complexity may reflect interaction between cerebellar gating and another evolutionary filtering system - polyvagal frequency modulation.

### 9.5 Integrating cerebellar sensory gating and polyvagal frequency filtering

The frequency-specific effects of mescaline on PPI may reflect a fundamental interaction between two evolutionary filtering systems - polyvagal acoustic frequency modulation and the cerebellar-brainstem gating circuits. Porges and Lewis demonstrated across mammalian species that the middle ear muscles, controlled by the ventral vagal system, act as frequency filters (100). In the case of humans, the frequency filter is optimized for voice prosody (approximately 500–4000 Hz) during states of safety and social engagement. When the autonomic nervous system shifts into defensive states, this filtering moves away from social frequencies toward lower frequencies associated with predator detection.

### 9.6 State-dependent frequency tuning

Critically, the polyvagal theory proposes that when individuals are in ventral vagal states of safety and social engagement, the stapedius and tensor tympani muscles actively dampen low-frequency background sounds while enhancing sensitivity to the social communication band, facilitating extraction of prosodic cues that signal safety. However, when the nervous system shifts into sympathetic mobilization or dorsal vagal shutdown, states characteristic of trauma, anxiety, and psychiatric disorders, the neural tone to these middle ear muscles is compromised. This results in a loss of frequency filtering optimization, i.e., individuals become hypervigilant to low-frequency threatening sounds (predator footsteps, rumbling) and high-frequency distress signals while simultaneously losing the ability to clearly perceive the mid-range frequencies that carry social safety cues in human voices. This state-dependent shift in frequency filtering may explain why individuals with PTSD, anxiety disorders, and other conditions struggle to hear reassurance or emotional nuance in others’ voices even when physically present - their middle ear muscles are literally tuned to detect threat rather than connection.

### 9.7 Integration with psychedelic effects

The differential effects observed in mescaline treated rats across frequencies, enhanced gating at 4 kHz and 20 kHz but impaired gating at the intermediate 12 kHz, suggest that psychedelics do not uniformly affect all acoustic filtering, but rather produces frequency-specific modulation of the gating circuitry. Notably, 12 kHz falls between the lower frequencies typically associated with environmental and predator sounds and the ultrasonic ranges where rats produce social vocalizations, potentially representing a transitional frequency band in the auditory processing hierarchy. This frequency-dependent dissociation suggests that mescaline may temporarily decouple rigid, state-dependent filtering patterns by differentially affecting distinct components of the cerebellar-brainstem gating system. This could represent a therapeutic mechanism. Selectively disrupting maladaptive frequency specific filtering while preserving or enhancing gating at other frequencies, psychedelics may create a window for recalibrating both the top-down vagal modulation of middle ear function and the bottom-up cerebellar gating of acoustic inputs. The result would be a restoration of flexible, context-appropriate filtering where acoustically salient information is properly gated based on actual environmental demands rather than trauma-conditioned hypervigilance. This integration suggests that effective psychedelic-assisted therapy may require consideration of the acoustic environment during treatment, potentially combining psychedelic-induced plasticity with strategic acoustic stimulation to re-establish healthy vagal-cerebellar filtering coordination across the full frequency spectrum relevant to safety and threat detection.

The interaction between polyvagal frequency filtering and cerebellar gating suggests that psychedelics may decouple rigid, trauma-conditioned filtering patterns. However, the acute session represents only the beginning of the therapeutic process. The lasting benefits of psychedelics derive from neuroplastic changes that unfold over days to weeks following administration.

## 10 Neuroplasticity mechanisms: beyond acute effects

The acute hyperconnectivity and altered filtering during a psychedelic session is only part of the story. Equally or more important are the lasting neuroplastic changes these drugs initiate. Preclinical studies have consistently demonstrated that psychedelics promote structural and functional neural plasticity, potentially explaining their enduring therapeutic effects.

### 10.1 Synaptogenesis and dendritic growth

Within hours of exposure to LSD, psilocybin, or DMT, cortical neurons show increased dendritic spine density and dendritic arborization (101). These are not subtle changes: spine density can increase by 10-20% within 24 hours, with effects persisting for days to weeks. The newly formed spines represent potential new synaptic connections—physical substrate for altered neural circuits. This burst of synaptogenesis appears to be mediated by 5-HT2A receptor activation leading to increased brain-derived neurotrophic factor (BDNF) expression, activation of the mammalian target of rapamycin (mTOR) pathway, and upregulation of plasticity-related genes (102). Remarkably, the magnitude of psychedelic-induced synaptogenesis is comparable to or greater than that produced by rapid-acting antidepressants like ketamine, which also promote spine growth through different molecular mechanisms (NMDA receptor antagonism). The anatomical distribution of this plasticity is important. While most studies have focused on cortical regions (particularly prefrontal cortex and hippocampus), evidence suggests that psychedelics may also promote plasticity in subcortical structures including the cerebellum. If psychedelics enhance synaptic plasticity in cerebellar circuits, this could allow for recalibration of filtering parameters through new synaptic configurations.

### 10.2 Critical period reopening

During early life, sensory and cognitive systems go through critical periods when the brain is especially malleable, allowing environmental input to shape circuit development. Sensory gating mechanisms are established and refined during these developmental windows, after which they typically become relatively stable and resistant to change in late childhood or adolescence. Nardou et al. demonstrated that psychedelics can reopen the social reward learning critical period in adult mice, suggesting a general mechanism by which these compounds restore developmental-like plasticity to mature neural circuits (103). Psychedelics may transiently reopen these windows of heightened plasticity in the adult brain. This would explain their ability to produce lasting changes in perception, cognition, and emotional patterns after even a single session.

### 10.3 The importance of timing

If filtering mechanisms are typically established during development and then become relatively fixed, a psychedelic-induced critical period reopening might allow those mechanisms to be fundamentally reset based on current experience and therapeutic input. The timing of therapeutic interventions may be critical. If psychedelics open a window of enhanced plasticity, then the experiences, therapy, and environmental inputs during and immediately after the psychedelic session could shape how circuits rewire. This aligns with clinical evidence suggesting that psychedelic-assisted psychotherapy, which pairs the drug with structured therapeutic support, produces better outcomes than drug administration alone. Critical period reopening explains how single psychedelic sessions can produce lasting changes, but it also reveals a vulnerability: if circuits become highly malleable, what determines whether they rewire adaptively or maladaptively? For disorders rooted in traumatic memories, this question becomes especially important.

### 10.4 Psychedelics and fear memory: relevance to PTSD

Numerous preclinical studies demonstrate that psychedelics can facilitate the extinction of fear memories and prevent the reconsolidation of aversive memories (104, 105). In rodent models of fear conditioning, psychedelics administered around the time of extinction training enhance extinction learning and reduce subsequent fear renewal. Similarly, when psychedelics are given during the reconsolidation window (shortly after memory reactivation), they can disrupt reconsolidation, leading to lasting attenuation of the fear memory. These effects have been documented with multiple psychedelic compounds and across various fear conditioning paradigms (auditory fear conditioning, contextual fear conditioning, fear-potentiated startle). The mechanisms appear to involve plasticity in prefrontal-amygdala-hippocampal circuits that mediate fear learning and extinction.

### 10.5 Dual mechanism: top-down and bottom-up

However, most discussions of these findings frame them within a top-down extinction learning framework. The implicit model is that psychedelics enhance cortically-mediated relearning of safety signals and suppression of fear responses. While this is certainly part of the mechanism, the cerebellar filtering framework suggests an additional, complementary process.

If psychedelics reset cerebellar filtering mechanisms, they might simultaneously enable both top-down extinction (cortically mediated) and bottom-up gating (cerebellum mediated). The acute disruption of filtering during the psychedelic session could allow trauma-associated stimuli to be re-processed in a new context, one where the person feels safe and supported. During the subsequent plasticity window, new associations can be formed while old ones are weakened. Critically, if the cerebellum’s filtering parameters are reset, trauma triggers might lose their ability to activate startle and arousal circuits in the first place, independent of cortical extinction. This dual mechanism, combined top-down extinction and bottom-up filter reset, could explain why psychedelic-assisted therapy for PTSD shows such promising early results in clinical trials, with effect sizes often larger than those seen with traditional exposure therapy or SSRI medications alone.

The effects on fear memory demonstrate psychedelics’ potential for PTSD, but they also reveal the importance of timing and context. The plasticity window is not indefinite—it opens acutely during and after the session, then gradually closes. This temporal dynamic leads to a two-phase model of how psychedelics produce therapeutic change.

## 11 Psychedelic mechanisms: from chaos to reorganization

How does the abrupt, chaotic effect of psychedelics translate into long-lasting enhanced filtering? This treatise proposes two phases to this reorganization beginning with an acute network collapse followed by reorganization of the network structure.

### 11.1 Phase 1: acute collapse of filtering (hours)

5-HT2A agonism on Purkinje cells and inferior olivary neurons disrupts the predictive cancellation mechanism. The cerebellum temporarily loses its ability to distinguish signal from noise. Consequently, there is massive cerebellar hyperconnectivity (as observed in our LSD, psilocybin, and mescaline studies) flooding of cortex with unfiltered information—both external sensory and internal cognitive. This is followed by effects like sensory intensification, synesthesia, ego dissolution, altered time perception—all consistent with the temporary failure of filters that normally organize and constrain conscious experience. This initial acute phase is not therapeutic in itself; it is the destabilization phase. The chaos is necessary but not sufficient.

### 11.2 Phase 2: post-acute reorganization (days to weeks)

The acute disruption triggers homeostatic plasticity mechanisms: synaptogenesis, dendritic remodeling, BDNF upregulation, mTOR activation. The cerebellum and its connected circuits enter a critical period-like state of enhanced plasticity. During this window, new environmental inputs can reshape filtering parameters. In a therapeutic setting (safe, supportive, with trauma processing), the system reorganizes around healthier filters. The traumatic stimulus loses its pathological salience; the rumination loop is broken. Without therapeutic context, reorganization may be random or even maladaptive—explaining why recreational use without integration support produces inconsistent outcomes (106). The mescaline/benzaldehyde finding noted above demonstrates recalibration: a previously hardwired salient stimulus became non-salient, suggesting filters were reset based on the drug state, not the stimulus history.

### 11.3 The critical importance of therapeutic context

The neuroplasticity induced by psychedelics is neutral—it simply makes circuits changeable. Whether those changes are therapeutic or harmful depends on the experiences, relationships, and processing that occur during the plasticity window. This is why indigenous traditions using psychedelics have always emphasized ritual, preparation, and integration, and why modern clinical trials showing the most impressive outcomes all include extensive therapeutic support before, during, and after drug administration.

The mescaline/benzaldehyde finding demonstrates this principle at the most basic level: a previously hardwired salient stimulus became non-salient, suggesting filters were reset based on the drug state and current environmental context, not the stimulus’s evolutionary history. The system learned new filtering parameters during the acute session, then consolidated those parameters during the post-acute plasticity window.

### 11.4 Mechanistic hypothesis

Psychedelics do not simply “reset” filters like rebooting a computer. Rather, they induce a controlled collapse of maladaptive filtering architectures followed by activity-dependent reorganization. The inferior olive-cerebellar circuit learns through prediction errors. Chronic psychiatric conditions may represent entrenched prediction models (“the world is dangerous” in PTSD). Psychedelics temporarily disable these models, forcing the system to rebuild predictions from current environmental input. The frequency-specific PPI effects we observed (enhanced gating at 4 and 20 kHz, impaired at 12 kHz) with mescaline suggest that reorganization is not global but frequency/modality-specific, potentially allowing targeted recalibration of particular filtering channels while leaving others intact.

The two-phase model explains both the promise and the peril of psychedelic therapy: acute disruption creates malleability, but therapeutic context determines whether reorganization is adaptive. The question for clinical practice becomes: how can we reliably harness this plasticity for therapeutic benefit across different psychiatric conditions, each with distinct filtering deficits?

## 12 Clinical translation: toward “noise-cancelling” therapeutics

The filtering framework reframes mental illness not as a disorder of brain chemistry per se, but as a disorder of information processing. Neurotransmitters like 5-HT, DA and NE retain their importance but are reconceptualized as regulators of filtering capacity rather than merely mood modulators. This perspective redirects therapeutic efforts from tweaking synaptic neurotransmitter concentrations to restoring the brain’s ability to automatically distinguish signal from noise.

All of these psychiatric disorders are not mutually exclusive. Many patients show deficits across multiple filtering types. Comorbidity between PTSD and depression, or between ADHD and anxiety, may reflect overlapping filtering dysfunctions affecting both external sensory and internal cognitive systems. As such, treatment should target the specific filtering deficit. Therapies effective for one filtering type may be ineffective or even counterproductive for another. Traditional exposure therapy for PTSD works through top-down extinction (Type 1 filtering enhancement) but may be less effective for depression (Type 2 filtering failure). Psychedelic-assisted therapy may reset filters across multiple types, explaining its transdiagnostic potential. Patients with severe PPI deficits (Type 1 dysfunction) might receive cerebellar neurostimulation + bottom-up sensory retraining. Those with intact PPI but altered cerebellar-prefrontal connectivity during negative processing (Type 2 dysfunction) might receive psychedelic-assisted therapy targeting internal filter recalibration. This moves psychiatry toward physiology-based rather than purely symptom-based treatment allocation. By distinguishing these filtering types and their associated neural circuits, the framework escapes the overgeneralization critique while retaining its core insight: psychiatric symptoms reflect failures of the brain’s evolved information processing architecture. The question is not whether filtering fails, but which filter, in which circuit, producing which symptoms.

### 12.1 Testable predictions and biomarkers

A valuable theory generates testable predictions. The filtering framework suggests several testable predictions:

1. Treatments that improve clinical symptoms in psychiatric disorders should also normalize sensory gating measures (PPI, P50 suppression). This can be tested longitudinally in clinical trials.
2. Baseline cerebellar-cortical connectivity should predict treatment response across different therapeutic modalities. Patients with more severe cerebellar connectivity disruption may require more intensive interventions.
3. Interventions that directly enhance cerebellar function (neurostimulation, cognitive training, physical exercise) should reduce psychiatric symptoms by improving filtering capacity.
4. Individual differences in cerebellar structure or function should predict vulnerability to developing psychiatric disorders following trauma or stress.
5. Psychedelic-induced changes in cerebellar connectivity should correlate with therapeutic outcomes, with patients showing the greatest connectivity normalization experiencing the best clinical response. These predictions are falsifiable and can be tested using existing neuroimaging and neurophysiological methods. Establishing PPI and P50 as clinical biomarkers would also provide objective outcome measures for treatment trials, complementing subjective symptom scales.

### 12.2 Alternative therapeutic approaches

If the cerebellum is a critical node in psychiatric pathophysiology, multiple approaches might target this structure:

#### 12.2.1 Transcranial magnetic stimulation

Cerebellar transcranial magnetic stimulation (TMS) has shown preliminary efficacy in small studies of schizophrenia and depression (107). Unlike prefrontal TMS, which is FDA-approved for depression, cerebellar TMS remains experimental. However, the filtering framework suggests it deserves more systematic investigation. Stimulation parameters would need optimization—different psychiatric conditions might benefit from different stimulation frequencies, intensities, or target locations within the cerebellum.

#### 12.2.2 Transcranial direct current stimulation

Cerebellar transcranial direct current stimulation (tDCS) modulates cerebellar excitability and has shown effects on motor learning and cognitive tasks in healthy individuals (108). Its therapeutic potential in psychiatric disorders is largely unexplored but represents a non-invasive, relatively low-cost intervention worth investigating.

#### 12.2.3 Vestibular stimulation

The vestibular system provides major inputs to the cerebellum. Caloric vestibular stimulation (irrigating the ear canal with warm or cold water) modulates cerebellar activity and has shown preliminary effects on mood and anxiety (109). More sophisticated vestibular stimulation protocols might offer a novel route to cerebellar modulation.

#### 12.2.4 Physical exercise and motor learning

Physical exercise, particularly activities requiring coordination and balance (dance, martial arts, gymnastics), robustly engages the cerebellum and promotes cerebellar plasticity (110). The well-documented antidepressant and anxiolytic effects of exercise may work partly through cerebellar mechanisms (111). Structured exercise interventions designed to maximally engage cerebellar circuits could be tested as adjunctive treatments.

#### 12.2.5 Safe and sound protocol™

A targeted acoustic intervention designed to engage the cochlear muscles and activate the social engagement system through prosodically modulated vocal input (112).

## 13 The cerebellar filtering framework and predictive processing theory

Computational psychiatry has long recognized that mental illness may reflect failures in predictive filtering. Predictive processing theory proposes that the brain is not a passive receiver of sensory information but an active prediction machine. Rather than processing the full sensory signal at each stage of the neural hierarchy, the brain continuously generates top-down predictions about expected inputs and transmits only the mismatch i.e., the prediction error upward for further processing. Inputs that conform to expectations are suppressed while only the unexpected demands computational resources. Friston’s free energy principle extended this idea into a unified mathematical framework in which perception, attention, learning, and action are all strategies for minimizing the brain’s surprise about its sensory states (113). Psychiatric filtering disorders are states of pathologically elevated free energy, because brains fail to minimize the surprise efficiently as subcortical gating mechanisms that should suppress predictable inputs have broken down. With prediction processing the brain can minimize free energy, reducing metabolic load by ensuring that only genuine prediction errors — signals that actually matter — reach the cortical hierarchy. Applied to psychiatry, this framework has proven particularly generative. Fletcher and Frith proposed that schizophrenic hallucinations arise from aberrant precision assigned to prediction errors (114). Adams and colleagues (115) formalized this as a pathological imbalance between top-down priors and bottom-up sensory precision (115). A productive counter-argument from Corlett and colleagues proposed instead that hallucinations reflect overly rigid top-down priors that generate perceptual content independently of sensory input (116), a debate between bottom-up error amplification and top-down prior inflexibility that remains unresolved and maps directly onto the distinction this treatise draws between Type 1 and Type 2 filtering dysfunction.

For all its explanatory power, predictive processing in its standard formulation leaves a critical question underspecified: where, anatomically, does automatic bottom-up sensory gating occur before information reaches the cortical hierarchy? The cerebellum provides the answer. Its unique architecture with 69 billion neurons performing massive parallel computation funneled through only three pairs of deep nuclei is precisely the structure natural selection would build to implement prediction error gating subcortically, prior to thalamic relay, and below the threshold of conscious awareness. The inferior olivary-cerebellar circuit instantiates the core predictive coding computation in concrete neural hardware: inferior olivary neurons fire when sensory reality deviates from expectation, transmitting prediction errors to Purkinje cells via climbing fibers, while the cerebellum’s existing predictive model, encoded in the mossy fiber-granule cell-parallel fiber pathway, adjusts inhibitory output to cancel expected inputs before they propagate further. When input matches prediction the gate closes; when it violates prediction the gate opens. This is predictive coding implemented in an identified subcortical circuit that operates automatically, continuously, and at minimal cortical cost — the energy-efficient bottom-up filtering architecture that **Section 5** argues evolution would strongly favor.

The cerebellar filtering framework is therefore best understood as a neuroanatomically specified extension of predictive processing theory, not a competitor to it. For psychedelic action specifically, Carhart-Harris and Friston’s (117) REBUS model proposing that psychedelics flatten the cortical hierarchy of precision-weighted priors by increasing the relative weight of bottom-up sensory signals (117), finds a subcortical mechanism in our neuroimaging findings: 5-HT2A receptor agonism on Purkinje cells and inferior olivary neurons disrupts the inferior olivary-cerebellar Great Gate, producing the dramatic cerebellar hyperconnectivity we observe under LSD, psilocybin, and mescaline (96, 97); (118), and propagating that disruption upward through the cerebello-thalamo-cortical pathway to destabilize the cortical precision hierarchy in exactly the manner REBUS describes. This integration carries an important implication for the present framework’s scientific standing: by proposing a specific circuit, a specific mechanism, and specific measurable predictions — whether the inferior olivary-cerebellar gate normalizes with effective treatment, whether cerebellar connectivity changes correlate with therapeutic outcome, whether psychedelic-induced cerebellar hyperconnectivity predicts clinical response — the cerebellar filtering framework inherits the explanatory scope of predictive processing while adding the anatomical specificity that renders it genuinely falsifiable.

## 14 Summary

This treatise proposes a paradigm shift in psychiatry: mental illness fundamentally represents a breakdown in the brain’s sensory filtering mechanisms rather than merely chemical imbalances at neurotransmitter receptors. After hundreds of millions of years of evolution shaped by the imperatives of finding food, securing mates, and avoiding predation, natural selection favored organisms capable of efficient, energy-conserving filtering, attending to survival-relevant stimuli while automatically suppressing irrelevant noise at brainstem and thalamic levels before engaging metabolically expensive cortical resources. Among sensory modalities, audition occupies a privileged evolutionary position as the “sentinel sense,” uniquely capable of omnidirectional threat detection and continuous operation during sleep when visual monitoring ceases, explaining why auditory gating deficits (measured via prepulse inhibition and P50 suppression) are particularly evident across psychiatric disorders.

The cerebellum emerges as the master filter implementing this bottom-up gating. Despite housing over 50% of the brain’s neurons, it funnels information through only three pairs of deep nuclei—creating an ideal architecture for distilling massive sensory input into refined control signals—positioned strategically between sensory input pathways (inferior and middle cerebellar peduncles) and cortical output (superior cerebellar peduncle). The inferior olivary-cerebellar “Great Gate” mechanism implements predictive coding, cancelling expected inputs as noise while amplifying unexpected signals. Three types of filtering dysfunction account for diverse psychiatric symptomatology: external sensory gating failures (schizophrenia, PTSD), internal cognitive/affective filtering deficits (depression, OCD), and contextual salience misweighting (autism, ADHD). Neurotransmitters serve as dynamic filtering regulators: serotonin (5-HT) adjusts global gating thresholds via Purkinje cell modulation, dopamine (DA) controls salience weighting through striato-cerebellar loops, and norepinephrine (NE) tunes arousal-dependent filtering sensitivity.

Recent neuroimaging reveals that psychedelic compounds (LSD, psilocybin, mescaline) may act as “reset buttons” for maladaptive filters through a unique therapeutic mechanism: acute 5-HT2A-mediated collapse of rigid filtering patterns producing dramatic cerebellar hyperconnectivity, followed by a post-acute plasticity window enabling activity-dependent reorganization of cerebellar-cortical circuits—including synaptogenesis, critical period reopening, and fear memory modification. Without therapeutic context, this reorganization may be random or even maladaptive, explaining why recreational use without integration support produces inconsistent outcomes, while structured psychedelic-assisted therapy yields more reliable therapeutic benefits. Translation to clinical practice requires multi-modal strategies combining pharmacological filter modulation (psychedelic-assisted therapy, cerebellar-targeted drugs), neuromodulation (cerebellar TMS/tDCS), behavioral training (coordination exercises, auditory gating protocols), and precision biomarker-guided treatment selection (PPI, P50, connectivity measures). This filtering framework provides testable predictions, objective biomarkers, and novel interventions for conditions that have resisted a half-century of receptor-focused drug development. The ultimate goal: restore the brain’s fundamental capacity to tune out noise and attend to meaning, allowing psychiatric patients to engage with their environment as adaptive organisms rather than overwhelmed systems—recognizing that when the brain’s most fundamental and evolutionarily conserved filtering capacity fails, organisms become overwhelmed by the ceaseless noise of existence, manifesting as the diverse symptomatology of psychiatric illness.

## Data Availability

Publicly available datasets were analyzed in this study. This data can be found here: PubMed.

## References

[1] Nutt DJ . Drug development in psychiatry: 50 years of failure and how to resuscitate it. Lancet Psychiatry. (2025) 12:228–38. doi: 10.1016/s2215-0366(24)00370-5. PMID: 39952266

[2] Ban TA . Fifty years chlorpromazine: a historical perspective. Neuropsychiatr Dis Treat. (2007) 3:495–500. 19300578 PMC2655089

[3] Hyman SE . Revitalizing psychiatric therapeutics. Neuropsychopharmacology. (2014) 39:220–9. doi: 10.1038/npp.2013.181. PMID: 24317307 PMC3857646

[4] Zhdanava M Pilon D Ghelerter I Chow W Joshi K Lefebvre P . The prevalence and national burden of treatment-resistant depression and major depressive disorder in the United States. J Clin Psychiatry. (2021) 82. doi: 10.4088/jcp.20m13699. PMID: 33989464

[5] Mcintyre RS Alsuwaidan M Baune BT Berk M Demyttenaere K Goldberg JF . Treatment-resistant depression: definition, prevalence, detection, management, and investigational interventions. World Psychiatry. (2023) 22:394–412. doi: 10.1002/wps.21120. PMID: 37713549 PMC10503923

[6] Spark DL Fornito A Langmead CJ Stewart GD . Beyond antipsychotics: A twenty-first century update for preclinical development of schizophrenia therapeutics. Transl Psychiatry. (2022) 12:147. doi: 10.1038/s41398-022-01904-2. PMID: 35393394 PMC8991275

[7] Akiki TJ Abdallah CG . Are there effective psychopharmacologic treatments for PTSD? J Clin Psychiatry. (2018) 80. doi: 10.4088/jcp.18ac12473. PMID: 30695292 PMC6436624

[8] Humphreys RK Ruxton GD . A review of thanatosis (death feigning) as an anti-predator behaviour. Behav Ecol Sociobiol. (2018) 72:22. doi: 10.1007/s00265-017-2436-8. PMID: 29386702 PMC5769822

[9] Anderson MC Floresco SB . Prefrontal-hippocampal interactions supporting the extinction of emotional memories: the retrieval stopping model. Neuropsychopharmacology. (2022) 47:180–95. doi: 10.1038/s41386-021-01131-1. PMID: 34446831 PMC8616908

[10] Liu Y Ye S Li XN Li WG . Memory trace for fear extinction: fragile yet reinforceable. Neurosci Bull. (2024) 40:777–94. doi: 10.1007/s12264-023-01129-3. PMID: 37812300 PMC11178705

[11] Plas SL Tuna T Bayer H Juliano VAL Sweck SO Arellano Perez AD . Neural circuits for the adaptive regulation of fear and extinction memory. Front Behav Neurosci. (2024) 18:1352797. doi: 10.3389/fnbeh.2024.1352797. PMID: 38370858 PMC10869525

[12] Sweet B Giovannetti D . Design of an eye limiting resolution visual system using commercial-off-the-shelf equipment. In: AIAA modeling and simulation technologies conference (2008).

[13] Carlini A Bordeau C Ambard M . Auditory localization: a comprehensive practical review. Front Psychol. (2024) 15:1408073. doi: 10.3389/fpsyg.2024.1408073. PMID: 39049946 PMC11267622

[14] Acerbi A Nunn C . Predation and the phasing of sleep: an evolutionary individual-based model. Anim Behav. (2011). doi: 10.1016/j.anbehav.2011.01.015. PMID: 41859675

[15] Aime M Adamantidis AR . Sleep to survive predators. Neurosci Bull. (2022) 38:1114–6. doi: 10.1007/s12264-022-00875-0. PMID: 35570232 PMC9468192

[16] Coenen A . Sensory gating and gaining in sleep: the balance between the protection of sleep and the safeness of life (a review). J Sleep Res. (2024). doi: 10.1111/jsr.14152. PMID: 38286435

[17] Claude L Chouchou F Prados G Castro M De Blay B Perchet C . Sleep spindles and human cortical nociception: a surface and intracerebral electrophysiological study. J Physiol. (2015) 593:4995–5008. doi: 10.1113/jp270941. PMID: 26377229 PMC4650416

[18] Jourde HR Coffey EBJ . Auditory processing up to cortex is maintained during sleep spindles. PNAS Nexus. (2024) 3:pgae479. doi: 10.1093/pnasnexus/pgae479. PMID: 39588317 PMC11586671

[19] Portas CM Krakow K Allen P Josephs O Armony JL Frith CD . Auditory processing across the sleep-wake cycle: simultaneous EEG and fMRI monitoring in humans. Neuron. (2000) 28:991–9. doi: 10.1016/s0896-6273(00)00169-0. PMID: 11163282

[20] Legendre GYT Moyne M Dominguez-Borras J Kumar S Sterpenich V Schwartz S . Scream’s roughness grants privileged access to the brain during sleep. Sci Rep. (2025) 15:16686. doi: 10.1038/s41598-025-01560-8. PMID: 40369048 PMC12078618

[21] Mayer AR Hanlon FM Franco AR Teshiba TM Thoma RJ Clark VP . The neural networks underlying auditory sensory gating. Neuroimage. (2009) 44:182–94. doi: 10.1016/j.neuroimage.2008.08.025. PMID: 18801443 PMC2656944

[22] Khani A Lanz F Loquet G Schaller K Michel C Quairiaux C . Large-scale networks for auditory sensory gating in the awake mouse. eNeuro. (2019) 6. doi: 10.1523/eneuro.0207-19.2019. PMID: 31444224 PMC6734044

[23] Gomez-Nieto R Horta-Junior Jde A Castellano O Millian-Morell L Rubio ME Lopez DE . Origin and function of short-latency inputs to the neural substrates underlying the acoustic startle reflex. Front Neurosci. (2014) 8:216. doi: 10.3389/fnins.2014.00216. PMID: 25120419 PMC4110630

[24] Braff DL Geyer MA Swerdlow NR . Human studies of prepulse inhibition of startle: normal subjects, patient groups, and pharmacological studies. Psychopharmacol (Berl). (2001) 156:234–58. doi: 10.1007/s002130100810. PMID: 11549226

[25] Freedman R Adler LE Waldo MC Pachtman E Franks RD . Neurophysiological evidence for a defect in inhibitory pathways in schizophrenia: comparison of medicated and drug-free patients. Biol Psychiatry. (1983) 18:537–51. 6134559

[26] Braff DL Light GA Swerdlow NR . Prepulse inhibition and P50 suppression are both deficient but not correlated in schizophrenia patients. Biol Psychiatry. (2007) 61:1204–7. doi: 10.1016/j.biopsych.2006.08.015. PMID: 17161386

[27] Holstein DH Vollenweider FX Jancke L Schopper C Csomor PA . P50 suppression, prepulse inhibition, and startle reactivity in the same patient cohort suffering from posttraumatic stress disorder. J Affect Disord. (2010) 126:188–97. doi: 10.1016/j.jad.2010.02.122. PMID: 20347156

[28] Hugdahl K Loberg EM Specht K Steen VM Van Wageningen H Jorgensen HA . Auditory hallucinations in schizophrenia: the role of cognitive, brain structural and genetic disturbances in the left temporal lobe. Front Hum Neurosci. (2007) 1:6.2007. doi: 10.3389/neuro.09.006.2007. PMID: 18958220 PMC2525988

[29] Danesh AA Howery S Aazh H Kaf W Eshraghi AA . Hyperacusis in autism spectrum disorders. Audiol Res. (2021) 11:547–56. doi: 10.3390/audiolres11040049. PMID: 34698068 PMC8544234

[30] Brout JJ Edelstein M Erfanian M Mannino M Miller LJ Rouw R . Investigating misophonia: a review of the empirical literature, clinical implications, and a research agenda. Front Neurosci. (2018) 12:36. doi: 10.3389/fnins.2018.00036. PMID: 29467604 PMC5808324

[31] Atagun MI Drukker M Hall MH Altun IK Tatli SZ Guloksuz S . Meta-analysis of auditory P50 sensory gating in schizophrenia and bipolar disorder. Psychiatry Res Neuroimaging. (2020) 300:111078. doi: 10.1016/j.pscychresns.2020.111078. PMID: 32361172

[32] Shen CL Chou TL Lai WS Hsieh MH Liu CC Liu CM . P50, N100, and P200 auditory sensory gating deficits in schizophrenia patients. Front Psychiatry. (2020) 11:868. doi: 10.3389/fpsyt.2020.00868. PMID: 33192632 PMC7481459

[33] Geyer MA . The family of sensorimotor gating disorders: comorbidities or diagnostic overlaps? Neurotox Res. (2006) 10:211–20. doi: 10.1007/bf03033358. PMID: 17197371 PMC2667105

[34] Nakajima M Schmitt LI Halassa MM . Prefrontal cortex regulates sensory filtering through a basal ganglia-to-thalamus pathway. Neuron. (2019) 103:445–58. doi: 10.1016/j.neuron.2019.05.026. PMID: 31202541 PMC6886709

[35] Gomez-Nieto R Hormigo S Lopez DE . Prepulse inhibition of the auditory startle reflex assessment as a hallmark of brainstem sensorimotor gating mechanisms. Brain Sci. (2020) 10. doi: 10.3390/brainsci10090639. PMID: 32947873 PMC7563436

[36] Attwell D Laughlin SB . An energy budget for signaling in the grey matter of the brain. J Cereb Blood Flow Metab. (2001) 21:1133–45. doi: 10.1097/00004647-200110000-00001. PMID: 11598490

[37] Raichle ME Gusnard DA . Appraising the brain’s energy budget. Proc Natl Acad Sci USA. (2002) 99:10237–9. doi: 10.1073/pnas.172399499. PMID: 12149485 PMC124895

[38] Azevedo FA Carvalho LR Grinberg LT Farfel JM Ferretti RE Leite RE . Equal numbers of neuronal and nonneuronal cells make the human brain an isometrically scaled-up primate brain. J Comp Neurol. (2009) 513:532–41. doi: 10.1002/cne.21974. PMID: 19226510

[39] Consalez GG Goldowitz D Casoni F Hawkes R . Origins, development, and compartmentation of the granule cells of the cerebellum. Front Neural Circuits. (2020) 14:611841. doi: 10.3389/fncir.2020.611841. PMID: 33519389 PMC7843939

[40] Rudolph S Badura A Lutzu S Pathak SS Thieme A Verpeut JL . Cognitive-affective functions of the cerebellum. J Neurosci. (2023) 43:7554–64. doi: 10.1523/jneurosci.1451-23.2023. PMID: 37940582 PMC10634583

[41] Schmahmann JD Sherman JC . The cerebellar cognitive affective syndrome. Brain. (1998) 121:561–79. doi: 10.1093/brain/121.4.561. PMID: 9577385

[42] Stoodley CJ Schmahmann JD . Functional topography in the human cerebellum: A meta-analysis of neuroimaging studies. Neuroimage. (2009) 44:489–501. doi: 10.1016/j.neuroimage.2008.08.039. PMID: 18835452

[43] Chen SH Ho MH Desmond JE . A meta-analysis of cerebellar contributions to higher cognition from PET and fMRI studies. Hum Brain Mapp. (2014) 35:593–615. doi: 10.1002/hbm.22194. PMID: 23125108 PMC3866223

[44] De Benedictis A Rossi-Espagnet MC De Palma L Carai A Marras CE . Networking of the human cerebellum: from anatomo-functional development to neurosurgical implications. Front Neurol. (2022) 13:806298. doi: 10.3389/fneur.2022.806298. PMID: 35185765 PMC8854219

[45] Devor A . The great gate: control of sensory information flow to the cerebellum. Cerebellum. (2002) 1:27–34. doi: 10.1080/147342202753203069. PMID: 12879971

[46] Palesi F Tournier JD Calamante F Muhlert N Castellazzi G Chard D . Contralateral cerebello-thalamo-cortical pathways with prominent involvement of associative areas in humans *in vivo*. Brain Struct Funct. (2015) 220:3369–84. doi: 10.1007/s00429-014-0861-2. PMID: 25134682 PMC4575696

[47] Strick PL Dum RP Fiez JA . Cerebellum and nonmotor function. Annu Rev Neurosci. (2009) 32:413–34. doi: 10.1146/annurev.neuro.31.060407.125606. PMID: 19555291

[48] Koziol LF Budding D Andreasen N D’arrigo S Bulgheroni S Imamizu H . Consensus paper: the cerebellum’s role in movement and cognition. Cerebellum. (2014) 13:151–77. doi: 10.1007/s12311-013-0511-x. PMID: 23996631 PMC4089997

[49] Middleton FA Strick PL . Anatomical evidence for cerebellar and basal ganglia involvement in higher cognitive function. Science. (1994) 266:458–61. doi: 10.1126/science.7939688. PMID: 7939688

[50] Kebschull JM Richman EB Ringach N Friedmann D Albarran E Kolluru SS . Cerebellar nuclei evolved by repeatedly duplicating a conserved cell-type set. Science. (2020) 370. doi: 10.1126/science.abd5059. PMID: 33335034 PMC8510508

[51] Ito M . Cerebellar circuitry as a neuronal machine. Prog Neurobiol. (2006) 78:272–303. doi: 10.1016/j.pneurobio.2006.02.006. PMID: 16759785

[52] Dean P Porrill J Ekerot CF Jorntell H . The cerebellar microcircuit as an adaptive filter: experimental and computational evidence. Nat Rev Neurosci. (2010) 11:30–43. doi: 10.1038/nrn2756. PMID: 19997115

[53] Hull C . Prediction signals in the cerebellum: beyond supervised motor learning. Elife. (2020) 9. doi: 10.7554/elife.54073. PMID: 32223891 PMC7105376

[54] Ito M . Cerebellar long-term depression: characterization, signal transduction, and functional roles. Physiol Rev. (2001) 81:1143–95. doi: 10.1152/physrev.2001.81.3.1143. PMID: 11427694

[55] Gao Z Van Beugen BJ De Zeeuw CI . Distributed synergistic plasticity and cerebellar learning. Nat Rev Neurosci. (2012) 13:619–35. doi: 10.1038/nrn3312. PMID: 22895474

[56] Chan-Palay V . Fine structure of labelled axons in the cerebellar cortex and nuclei of rodents and primates after intraventricular infusions with tritiated serotonin. Anat Embryol (Berl). (1975) 148:235–46. doi: 10.1007/bf00319846. PMID: 816225

[57] Takeuchi Y Kimura H Sano Y . Immunohistochemical demonstration of serotonin-containing nerve fibers in the cerebellum. Cell Tissue Res. (1982) 226:1–12. doi: 10.1007/bf00217077. PMID: 6751545

[58] Bishop GA Ho RH . The distribution and origin of serotonin immunoreactivity in the rat cerebellum. Brain Res. (1985) 331:195–207. doi: 10.1016/0006-8993(85)91545-8. PMID: 3986565

[59] Strahlendorf JC Strahlendorf HK Lee M . Enhancement of cerebellar Purkinje cell complex discharge activity by microiontophoretic serotonin. Exp Brain Res. (1986) 61:614–24. doi: 10.1007/bf00237588. PMID: 3956618

[60] Dieudonne S . Serotonergic neuromodulation in the cerebellar cortex: cellular, synaptic, and molecular basis. Neuroscientist. (2001) 7:207–19. doi: 10.1177/107385840100700306, PMID: 11499400

[61] Maeshima T Shutoh F Hamada S Senzaki K Hamaguchi-Hamada K Ito R . Serotonin2A receptor-like immunoreactivity in rat cerebellar Purkinje cells. Neurosci Lett. (1998) 252:72–4. doi: 10.1016/s0304-3940(98)00546-1. PMID: 9756362

[62] Oostland M Buijink MR Teunisse GM Von Oerthel L Smidt MP Van Hooft JA . Distinct temporal expression of 5-HT(1A) and 5-HT(2A) receptors on cerebellar granule cells in mice. Cerebellum. (2014) 13:491–500. doi: 10.1007/s12311-014-0565-4. PMID: 24788088 PMC4077297

[63] Best AR Regehr WG . Serotonin evokes endocannabinoid release and retrogradely suppresses excitatory synapses. J Neurosci. (2008) 28:6508–15. doi: 10.1523/jneurosci.0678-08.2008. PMID: 18562622 PMC2720684

[64] Paterson DS Darnall R . 5-HT2A receptors are concentrated in regions of the human infant medulla involved in respiratory and autonomic control. Auton Neurosci. (2009) 147:48–55. doi: 10.1016/j.autneu.2009.01.004. PMID: 19213611 PMC2699562

[65] Vollenweider FX Preller KH . Psychedelic drugs: Neurobiology and potential for treatment of psychiatric disorders. Nat Rev Neurosci. (2020) 21:611–24. doi: 10.1038/s41583-020-0367-2. PMID: 32929261

[66] Siegel JS Subramanian S Perry D Kay BP Gordon EM Laumann TO . Psilocybin desynchronizes the human brain. Nature. (2024) 632:131–8. doi: 10.1038/s41586-024-07624-5. PMID: 39020167 PMC11291293

[67] Hoshi E Tremblay L Feger J Carras PL Strick PL . The cerebellum communicates with the basal ganglia. Nat Neurosci. (2005) 8:1491–3. doi: 10.1038/nn1544. PMID: 16205719

[68] Bostan AC Dum RP Strick PL . Cerebellar networks with the cerebral cortex and basal ganglia. Trends Cognit Sci. (2013) 17:241–54. doi: 10.1016/j.tics.2013.03.003. PMID: 23579055 PMC3645327

[69] Carta I Chen CH Schott AL Dorizan S Khodakhah K . Cerebellar modulation of the reward circuitry and social behavior. Science. (2019) 363. doi: 10.1126/science.aav0581. PMID: 30655412 PMC6711161

[70] Quartarone A Cacciola A Milardi D Ghilardi MF Calamuneri A Chillemi G . New insights into cortico-basal-cerebellar connectome: clinical and physiological considerations. Brain. (2020) 143:396–406. doi: 10.1093/brain/awz310. PMID: 31628799

[71] Kapur S . Psychosis as a state of aberrant salience: a framework linking biology, phenomenology, and pharmacology in schizophrenia. Am J Psychiatry. (2003) 160:13–23. doi: 10.1176/appi.ajp.160.1.13. PMID: 12505794

[72] Howes OD Mccutcheon R Owen MJ Murray RM . The role of genes, stress, and dopamine in the development of schizophrenia. Biol Psychiatry. (2017) 81:9–20. doi: 10.1016/j.biopsych.2016.07.014. PMID: 27720198 PMC5675052

[73] Mccutcheon RA Abi-Dargham A Howes OD . Schizophrenia, dopamine and the striatum: from biology to symptoms. Trends Neurosci. (2019) 42:205–20. doi: 10.1016/j.tins.2018.12.004. PMID: 30621912 PMC6401206

[74] Kapur S Remington G . Dopamine D(2) receptors and their role in atypical antipsychotic action: still necessary and may even be sufficient. Biol Psychiatry. (2001) 50:873–83. doi: 10.1016/s0006-3223(01)01251-3. PMID: 11743942

[75] Reyes BAS . The locus coeruleus: Anatomy, physiology, and stress-related neuropsychiatric disorders. Eur J Neurosci. (2025) 61:e70111. doi: 10.1111/ejn.70111. PMID: 40219735 PMC11992612

[76] Aston-Jones G Cohen JD . An integrative theory of locus coeruleus-norepinephrine function: adaptive gain and optimal performance. Annu Rev Neurosci. (2005) 28:403–50. doi: 10.1146/annurev.neuro.28.061604.135709. PMID: 16022602

[77] Naegeli C Zeffiro T Piccirelli M Jaillard A Weilenmann A Hassanpour K . Locus coeruleus activity mediates hyperresponsiveness in posttraumatic stress disorder. Biol Psychiatry. (2018) 83:254–62. doi: 10.1016/j.biopsych.2017.08.021. PMID: 29100627

[78] Raskind MA Peterson K Williams T Hoff DJ Hart K Holmes H . A trial of prazosin for combat trauma PTSD with nightmares in active-duty soldiers returned from Iraq and Afghanistan. Am J Psychiatry. (2013) 170:1003–10. doi: 10.1176/appi.ajp.2013.12081133. PMID: 23846759

[79] Guo W Liu F Chen J Wu R Zhang Z Yu M . Resting-state cerebellar-cerebral networks are differently affected in first-episode, drug-naive schizophrenia patients and unaffected siblings. Sci Rep. (2015) 5:17275. doi: 10.1038/srep17275. PMID: 26608842 PMC4660304

[80] Bang M Park HJ Pae C Park K Lee E Lee SK . Aberrant cerebro-cerebellar functional connectivity and minimal self-disturbance in individuals at ultra-high risk for psychosis and with first-episode schizophrenia. Schizophr Res. (2018) 202:138–40. doi: 10.1016/j.schres.2018.06.031. PMID: 29925474

[81] Moberget T Doan NT Alnaes D Kaufmann T Cordova-Palomera A Lagerberg TV . Cerebellar volume and cerebellocerebral structural covariance in schizophrenia: a multisite mega-analysis of 983 patients and 1349 healthy controls. Mol Psychiatry. (2018) 23:1512–20. doi: 10.1038/mp.2017.106. PMID: 28507318

[82] Ji JL Diehl C Schleifer C Tamminga CA Keshavan MS Sweeney JA . Schizophrenia exhibits bi-directional brain-wide alterations in cortico-striato-cerebellar circuits. Cereb Cortex. (2019) 29:4463–87. doi: 10.1093/cercor/bhy306. PMID: 31157363 PMC6917525

[83] Xie YJ Xi YB Cui LB Guan MZ Li C Wang ZH . Functional connectivity of cerebellar dentate nucleus and cognitive impairments in patients with drug-naive and first-episode schizophrenia. Psychiatry Res. (2021) 300:113937. doi: 10.1016/j.psychres.2021.113937. PMID: 33895443

[84] Andreasen NC O’leary DS Cizadlo T Arndt S Rezai K Ponto LL . Schizophrenia and cognitive dysmetria: a positron-emission tomography study of dysfunctional prefrontal-thalamic-cerebellar circuitry. Proc Natl Acad Sci USA. (1996) 93:9985–90. doi: 10.1073/pnas.93.18.9985. PMID: 8790444 PMC38542

[85] Mccarthy-Jones S Smailes D Corvin A Gill M Morris DW Dinan TG . Occurrence and co-occurrence of hallucinations by modality in schizophrenia-spectrum disorders. Psychiatry Res. (2017) 252:154–60. doi: 10.1016/j.psychres.2017.01.102. PMID: 28273630

[86] Kumari V Peters ER Fannon D Premkumar P Aasen I Cooke MA . Uncontrollable voices and their relationship to gating deficits in schizophrenia. Schizophr Res. (2008) 101:185–94. doi: 10.1016/j.schres.2007.12.481. PMID: 18262774 PMC2845800

[87] Kearney B Densmore M Théberge J Jetly R Mckinnon M Shaw S . Reduced cerebello-thalamo-cortical functional connectivity during traumatic memory retrieval in PTSD. Nat Ment Health. (2025). doi: 10.1038/s44220-025-00476-6. PMID: 41851180

[88] Liu L Zeng LL Li Y Ma Q Li B Shen H . Altered cerebellar functional connectivity with intrinsic connectivity networks in adults with major depressive disorder. PloS One. (2012) 7:e39516. doi: 10.1371/journal.pone.0039516. PMID: 22724025 PMC3377654

[89] Ludewig S Ludewig K . No prepulse inhibition deficits in patients with unipolar depression. Depress Anxiety. (2003) 17:224–5. doi: 10.1002/da.10109. PMID: 12820179

[90] Quednow BB Westheide J Kuhn KU Werner P Maier W Hawellek B . Normal prepulse inhibition and habituation of acoustic startle response in suicidal depressive patients without psychotic symptoms. J Affect Disord. (2006) 92:299–303. doi: 10.1016/j.jad.2006.01.022. PMID: 16503358

[91] Kohl S Heekeren K Klosterkotter J Kuhn J . Prepulse inhibition in psychiatric disorders--apart from schizophrenia. J Psychiatr Res. (2013) 47:445–52. doi: 10.1016/j.jpsychires.2012.11.018. PMID: 23287742

[92] Hou W Cheng R Zhao Z Liao H Li J . Atypical and variable attention patterns reveal reduced contextual priors in children with autism spectrum disorder. Autism Res. (2024) 17:1572–1585. 10.1002/aur.3194 38975627

[93] Aday JS Wood JR Bloesch EK Davoli CC . Psychedelic drugs and perception: a narrative review of the first era of research. Rev Neurosci. (2021) 32:559–71. doi: 10.1515/revneuro-2020-0094. PMID: 33550787

[94] Holze F Ley L Muller F Becker AM Straumann I Vizeli P . Direct comparison of the acute effects of lysergic acid diethylamide and psilocybin in a double-blind placebo-controlled study in healthy subjects. Neuropsychopharmacology. (2022) 47:1180–7. doi: 10.1038/s41386-022-01297-2. PMID: 35217796 PMC9018810

[95] Kwan AC Olson DE Preller KH Roth BL . The neural basis of psychedelic action. Nat Neurosci. (2022) 25:1407–19. doi: 10.1038/s41593-022-01177-4. PMID: 36280799 PMC9641582

[96] Ghaw A Chunduri A Chang A Ortiz RJ Kozlowska M Kulkarni PP . Dose-dependent LSD effects on cortical/thalamic and cerebellar activity: brain oxygen level-dependent fMRI study in awake rats. Brain Commun. (2024) 6:fcae194. doi: 10.1093/braincomms/fcae194. PMID: 38863575 PMC11166175

[97] Fuini E Chang A Ortiz RJ Nasseef T Edwards J Latta M . Dose-dependent changes in global brain activity and functional connectivity following exposure to psilocybin: a BOLD MRI study in awake rats. Front Neurosci. (2025) 19:1554049. doi: 10.3389/fnins.2025.1554049. PMID: 40376612 PMC12078138

[98] Bozza T Feinstein P Zheng C Mombaerts P . Odorant receptor expression defines functional units in the mouse olfactory system. J Neurosci. (2002) 22:3033–43. doi: 10.1523/jneurosci.22-08-03033.2002. PMID: 11943806 PMC6757547

[99] Kulkarni P Stolberg T Sullivanjr JM Ferris CF . Imaging evolutionarily conserved neural networks: preferential activation of the olfactory system by food-related odor. Behav Brain Res. (2012) 230:201–7. doi: 10.1016/j.bbr.2012.02.002. PMID: 22343130

[100] Porges S Lewis G . The polyvagal hypothesis: Common mechanisms mediating autonomic regulation, vocalizations and listening. In: Brudzynski SM , editor.Handbook of mammalian vocalizations: an integrative neuroscience approach (2010). p. 255–64.

[101] Ly C Greb AC Cameron LP Wong JM Barragan EV Wilson PC . Psychedelics promote structural and functional neural plasticity. Cell Rep. (2018) 23:3170–82. doi: 10.1016/j.celrep.2018.05.022. PMID: 29898390 PMC6082376

[102] Aleksandrova LR Phillips AG . Neuroplasticity as a convergent mechanism of ketamine and classical psychedelics. Trends Pharmacol Sci. (2021) 42:929–42. doi: 10.1016/j.tips.2021.08.003. PMID: 34565579

[103] Nardou R Sawyer E Song YJ Wilkinson M Padovan-Hernandez Y De Deus JL . Psychedelics reopen the social reward learning critical period. Nature. (2023) 618:790–8. doi: 10.1038/s41586-023-06204-3. PMID: 37316665 PMC10284704

[104] Doss MK Demarco A Dunsmoor JE Cisler JM Fonzo GA Nemeroff CB . How psychedelics modulate multiple memory mechanisms in posttraumatic stress disorder. Drugs. (2024) 84:1419–43. doi: 10.1007/s40265-024-02106-4. PMID: 39455547

[105] Werle I Bertoglio LJ . Psychedelics: A review of their effects on recalled aversive memories and fear/anxiety expression in rodents. Neurosci Biobehav Rev. (2024) 167:105899. doi: 10.1016/j.neubiorev.2024.105899. PMID: 39305969

[106] Brennan W Belser AB . Models of psychedelic-assisted psychotherapy: a contemporary assessment and an introduction to EMBARK, a transdiagnostic, trans-drug model. Front Psychol. (2022) 13:866018. doi: 10.3389/fpsyg.2022.866018. PMID: 35719571 PMC9201428

[107] Ciricugno A Paterno S Barbati N Borgatti R Cattaneo Z Ferrari C . Advances in cerebellar TMS therapy: an updated systematic review on multi-session interventions. Biomedicines. (2025) 13. doi: 10.3390/biomedicines13071578. PMID: 40722656 PMC12292720

[108] Ferrucci R Priori A . Transcranial cerebellar direct current stimulation (tcDCS): motor control, cognition, learning and emotions. Neuroimage. (2014) 85 Pt 3:918–23. doi: 10.1016/j.neuroimage.2013.04.122. PMID: 23664951

[109] Preuss N Hasler G Mast FW . Caloric vestibular stimulation modulates affective control and mood. Brain Stimul. (2014) 7:133–40. doi: 10.1016/j.brs.2013.09.003. PMID: 24139868

[110] Balazova Z Marecek R Novakova L Nemcova-Elfmarkova N Kropacova S Brabenec L . Dance intervention impact on brain plasticity: a randomized 6-month fMRI study in non-expert older adults. Front Aging Neurosci. (2021) 13:724064. doi: 10.3389/fnagi.2021.724064. PMID: 34776925 PMC8579817

[111] Wegner M Helmich I MaChado S Nardi AE Arias-Carrion O Budde H . Effects of exercise on anxiety and depression disorders: Review of meta- analyses and neurobiological mechanisms. CNS Neurol Disord Drug Targets. (2014) 13:1002–14. doi: 10.2174/1871527313666140612102841. PMID: 24923346

[112] Porges SW . Polyvagal theory: a journey from physiological observation to neural innervation and clinical insight. Front Behav Neurosci. (2025) 19:1659083. doi: 10.3389/fnbeh.2025.1659083. PMID: 41035859 PMC12479538

[113] Friston K . The free-energy principle: a unified brain theory? Nat Rev Neurosci. (2010) 11:127–38. doi: 10.1038/nrn2787. PMID: 20068583

[114] Fletcher PC Frith CD . Perceiving is believing: a Bayesian approach to explaining the positive symptoms of schizophrenia. Nat Rev Neurosci. (2009) 10:48–58. doi: 10.1038/nrn2536. PMID: 19050712

[115] Adams RA Stephan KE Brown HR Frith CD Friston KJ . The computational anatomy of psychosis. Front Psychiatry. (2013) 4:47. doi: 10.3389/fpsyt.2013.00047. PMID: 23750138 PMC3667557

[116] Corlett PR Horga G Fletcher PC Alderson-Day B Schmack K Powers A . Hallucinations and strong priors. Trends Cognit Sci. (2019) 23:114–27. doi: 10.1016/j.tics.2018.12.001. PMID: 30583945 PMC6368358

[117] Carhart-Harris RL Friston KJ . REBUS and the anarchic brain: toward a unified model of the brain action of psychedelics. Pharmacol Rev. (2019) 71:316–44. doi: 10.1124/pr.118.017160. PMID: 31221820 PMC6588209

[118] Cavallaro N Rai P Akins D Soltanpour S Nasseef T Ortiz RJ . Mescaline alters cerebellar function, global connectivity, and frequency-selective acoustic gating:. A BOLD fMRI study in awake rats Neurosci Bull (2026). 10.1007/s12264-026-01632-3 PMC13642855 42168715

